# SSS-test: a novel test for detecting positive selection on RNA secondary structure

**DOI:** 10.1186/s12859-019-2711-y

**Published:** 2019-03-21

**Authors:** Maria Beatriz Walter Costa, Christian Höner zu Siederdissen, Marko Dunjić, Peter F. Stadler, Katja Nowick

**Affiliations:** 1 Embrapa Agroenergia, Parque Estação Biológica (PqEB), Asa Norte, Brasília, DF, 70770-901 Brazil; 2 Bioinformatics Group, Department of Computer Science, and Interdisciplinary Center for Bioinformatics, Universität Leipzig, Härtelstraße 16–18, Leipzig, 04107 Germany; 3 Human Biology Group, Institute for Biology, Department of Biology, Chemistry, Pharmacy, Freie Universitaet Berlin, Königin-Luise-Straße 1-3, Berlin, 14195 Germany; 4 Center for Human Molecular Genetics, Faculty of Biology, University of Belgrade, Studentski trg 16, PO box 43, Belgrade, 11000 Serbia; 5 German Centre for Integrative Biodiversity Research (iDiv) Halle-Jena-Leipzig & Competence Center for Scalable Data Services and Solutions Dresden-Leipzig & Leipzig Research Center for Civilization Diseases, University Leipzig, Leipzig, 04107 Germany; 6 Max Planck Institute for Mathematics in the Sciences, Inselstraße 22, Leipzig, 04103 Germany; 7 Department of Theoretical Chemistry, University of Vienna, Währinger Straße 17, Vienna, A-1090 Austria; 8 Center for non-coding RNA in Technology and Health, University of Copenhagen, Grønnegårdsvej 3, Frederiksberg C, DK-1870 Denmark; 9 Faculdad de Ciencias, Universidad Nacional de Colombia, Sede Bogotá, Ciudad Universitaria, Bogotá, D.C., COL-111321 Colombia; 10 Santa Fe Institute, 1399 Hyde Park Rd., Santa Fe, NM87501 USA; 11 TFome Research Group, Bioinformatics Group, Interdisciplinary Center of Bioinformatics, Department of Computer Science, University of Leipzig, Härtelstraße 16-18, Leipzig, 04107 Germany; 12 Paul-Flechsig-Institute for Brain Research, University of Leipzig, Liebigstraße 19. Haus C, Leipzig, 04103 Germany; 13 Bioinformatics, Faculty of Agricultural Sciences, Institute of Animal Science, University of Hohenheim, Garbenstraße 13, Stuttgart, 70593 Germany

**Keywords:** Long non-coding RNA, Positive selection, RNA secondary structure, Psychiatric disorders, Primate genomes

## Abstract

**Background:**

Long non-coding RNAs (lncRNAs) play an important role in regulating gene expression and are thus important for determining phenotypes. Most attempts to measure selection in lncRNAs have focused on the primary sequence. The majority of small RNAs and at least some parts of lncRNAs must fold into specific structures to perform their biological function. Comprehensive assessments of selection acting on RNAs therefore must also encompass structure. Selection pressures acting on the structure of non-coding genes can be detected within multiple sequence alignments. Approaches of this type, however, have so far focused on negative selection. Thus, a computational method for identifying ncRNAs under positive selection is needed.

**Results:**

We introduce the SSS-test (test for Selection on Secondary Structure) to identify positive selection and thus adaptive evolution. Benchmarks with biological as well as synthetic controls yield coherent signals for both negative and positive selection, demonstrating the functionality of the test. A survey of a lncRNA collection comprising 15,443 families resulted in 110 candidates that appear to be under positive selection in human. In 26 lncRNAs that have been associated with psychiatric disorders we identified local structures that have signs of positive selection in the human lineage.

**Conclusions:**

It is feasible to assay positive selection acting on RNA secondary structures on a genome-wide scale. The detection of human-specific positive selection in lncRNAs associated with cognitive disorder provides a set of candidate genes for further experimental testing and may provide insights into the evolution of cognitive abilities in humans.

**Availability:**

The SSS-test and related software is available at: https://github.com/waltercostamb/SSS-test. The databases used in this work are available at: http://www.bioinf.uni-leipzig.de/Software/SSS-test/.

**Electronic supplementary material:**

The online version of this article (10.1186/s12859-019-2711-y) contains supplementary material, which is available to authorized users.

## Background

More than a decade of high-throughput transcriptomics has established wide-spread, pervasive transcription of mammalian genomes as an indisputable fact [1–5]. However, less than a quarter of the total RNA (excluding ribosomal RNAs) accounts for the about 19 000 protein coding genes and their isoforms [6]. The majority of the human transcriptome, in terms of diversity of the products, is composed of other, non-protein-coding, RNAs. These include small non-coding RNAs (ncRNAs) accounting for nearly the same genomic coverage as ORFs [7, 8], mRNA-like long non-coding RNAs (lncRNAs), as well as giant macroRNAs [5, 8, 9]. The current rather conservative estimate predicts 40 000 to 50 000 human lncRNA genes [10].

Although lncRNAs comprise a substantial fraction of the transcriptome, so far only a small minority of them has been assigned a functional annotation. The question thus remains, what fraction of the detectable lncRNAs actually convey biological functions, as opposed to being coherently transcribed and processed byproducts without biological relevance (“junk RNA”). Without experimental testing this question is currently difficult to answer because, in contrast to their protein-coding counterparts, most lncRNAs exhibit only low levels of sequence conservation. From a population genetics point of view, this relatively low level of sequence conservation is interpreted as lack of functional constraints or negative selection [11, 12]. However, as a group, lncRNAs do show signs of negative selection: for example, the cumulative distributions of substitution and transversion rates shows significantly suppressed values relative to neutrally evolving DNA [13], see also [2, 12, 14–17]. Furthermore, while the overall sequence conservation is low, gene structure and splice sites often seem to be highly conserved [18, 19], strongly suggesting that many lncRNAs are evolutionarily old [20–23]. Recent studies also found that lncRNAs are often located in syntenic positions and display similar expression patterns across species [24–26].

In this contribution we are concerned with the identification and quantification of selective pressures on RNA secondary structures. This is by no means a novel topic. From a population genetics point of view, two locus models have been used to study compensatory mutations, i.e., negative and stabilizing selection on RNA structures [27–29]. This line of studies showed that tRNAs are among the molecules with strongest selective pressures [30] and confirmed the influence of the effective population size as a cause of differences in selective constraints on tRNAs across species [31]. Altogether, thousands of well-studied small ncRNAs, mostly compiled in the Rfam [32] and miRBase [33] database, exhibit well-conserved, often nearly immutable, RNA secondary structures that are crucial for the function of the RNA molecule.

It is important to distinguish between the presence of conserved RNA secondary structure and signatures of selection on secondary structure. A conserved structure implies that only small deviations around a well-defined consensus structure are tolerated. In this case one expects sequence variation to have generated a sufficient number of compensatory mutations to test directly for the preservation of the consensus structure. R-scape [34] implements such a method. LncRNAs rarely, if ever, exhibit evidence for this level of structural conservation [34]. This seems to be limited to the “classical” families of small, structured ncRNAs and structured regulatory elements, i.e., the content of Rfam – and even these RNAs at times may show extensive structural variation, see e.g. [35, 36] and the references therein.

Negative selection on secondary structure, on the other hand, is a much less stringent property, and it suffices to show that structural variation is more constrained than what would be expected from the observed, underlying sequence variation. This idea has been used in a series of tools including qrna [37], AlifoldZ [38], EvoFold [39], CMfinder [40], RNAz [41], and SISSIz [42]. Extensive surveys [41, 43–47] of mammalian genomes already compiled evidence that a sizeable fraction of the human genome, possibly as much as 10% of the non-repetitive sequence, is under negative selection on RNA secondary structures. Intriguingly, these studies show that the genomic sequence from which a ncRNA is transcribed often evolves very rapidly, while still showing clear signs of selective constraints on local RNA secondary structures.

While the majority of the human lncRNA sequences evolve at average rates close to the unconstrained background, they show strong evidence for conservation of their gene structure, i.e., the preservation of splice sites, across the eutheria or even deeper phylogenetic groups [22]. It is not surprising therefore, that constrained structural modules have also been reported for some of the best studied examples [48–52]. The selective constraints are not strong enough, however, to enforce large, well-conserved consensus structures [34]. Further evidence for the importance of secondary structure features for the function of lncRNAs comes from disease-related SNPs [53, 54].

In contrast to negative selection on RNA secondary structure, very little is known about positive selection in this context beyond a single, well-studied example that might have a positively selected structure: Rapid, lineage specific changes of the sequence have been reported for the Human Accelerated Region 1 (HAR1). 18 human specific single-nucleotide substitutions in an element that is extremely conserved across non-human mammals make HAR1 the fastest evolving region in the human genome [55]. Interestingly, the human HAR1 forms a stable structure, which differs significantly from the chimpanzee structure of HAR1 [56–58]. Expression patterns of HAR1 suggest that it is involved in the development of the cortex [55]. However, real functional data for HAR1 is still missing. In particular, there is still no direct experimental proof that the function of HAR1 depends on its secondary structure. It is conceivable, therefore, that HAR1 acts based on its sequence, and that this function was human specifically lost. Nevertheless, examples such as HAR1 suggest that lncRNAs can potentially play an important role in species evolution.

To-date, no method is available to detect positive selection on RNA secondary structure in a systematic manner. It is an open question therefore, whether this is a rare phenomenon or whether positive selection on RNA structure is an important contributor to lineage-specific adaptation. In principle, positive selection can be identified by comparing the observed divergence with the expectation for neutral evolution. The *K*_*a*_/*K*_*s*_ test for coding sequences may serve as the paradigmatic example. The ratio of *K*_*a*_, the number of non-synonymous substitutions per non-synonymous sites, and *K*_*s*_, the number of synonymous substitutions per synonymous sites is expected to be larger than 1 when positive selection is acting (see e.g. [59]). This idea, however, does not generalize to non-coding RNAs since there is no analogous distinction between synonymous and non-synonymous substitutions.

An interesting alternative approach is to contrast more generic parameters of divergence and diversity between a functional element and a reference locus in its genomic vicinity. Plausible parameters are e.g. *ρ*, the fraction of sites under selection, the polymorphism rate *λ*, and the divergence rate *η* – in each case normalized by the corresponding parameter in the neutral control [60, 61]. These measures have been applied mostly at the level of groups of loci, which showed strong evidence that regulatory elements are influenced by selective pressures [62, 63].

As for any type of test for selection, an estimate of an effect on the phenotype is desired. As reviewed in [64], many tools have been developed in the past years contributing to unravelling the molecular mechanisms underlying complex phenotypes. Still, the effect of, say indels (insertions or deletions) and structural variation, remain elusive. If secondary structure is important for the function of a ncRNA, a predicted structural change can be taken as proxy for a phenotypic impact. The accumulation of substitutions that change the structure can be interpreted as signs of positive selection, or adaptive evolution.

Several methods have been proposed to quantify the effect of SNPs on RNA structures [65, 66]. Leveraging these methods, we propose here to use an excess of structure changing substitutions as a means of identifying positive selection. Conversely, an excess of substitutions that change the structure less than expected supports negative selection. We use this simple idea, while also accounting for the structural impact of insertions and deletions, to develop a statistical test for lineage-specific positive selection, the SSS-test (“Selection on the Secondary Structure test”). We then use this approach to identify candidate lncRNAs that might have been positively selected on the human lineage relative to their primate background. Among them are genes linked to psychiatric disorders (PDs) to provide further candidates that might have been involved in the evolution of the human brain.

## Theory

### SSS-test

The basic idea of the SSS-test is to determine whether selection pressures have changed in a particular lineage. The starting point for that is a multiple sequence alignment $\mathcal {A}$ of orthologous sequences of the RNA gene or element taken from a set of species under consideration. The SSS-test singles out one species, and hence one focal sequence $x \in \mathcal {A}$, and checks whether there is evidence of a change in the selection pressures acting on *x* compared to rest of the alignment $\mathcal {A}$. In order to assess how *x* is different, we consider the input alignment $\mathcal {A}$ with the focal sequence removed. This alignment $\mathcal {\bar A}=\mathcal {A}\setminus x$, serves as the background. Since the effect of variations can only be computed for individual sequences, we will also need the consensus sequences *z* of $\mathcal {A}$ and $\bar z$ of $\mathcal {\bar A}$. Note that $\bar z$, like $\mathcal {\bar A}$, depends on the focal sequence *x*. We do not indicate this dependence explicitly in the notation since it is clear from the context throughout. The idea of the SSS-test is to determine whether the effect of the individual changes leading from the background consensus $\bar z$ to the focal sequence change the secondary structure of $\bar z$ more than expected. To this end, we need to identify those sequence changes that set the focal sequence *x* apart from the background $\mathcal {\bar A}$ and its consensus $\bar z$.

Since we are interested in testing for lineage-specific positive selection, we consider only sites (alignment columns) *i* that are well-conserved in the background $\mathcal {\bar A}$. In other words, we need to exclude highly variable sites, because these convey no accessible information on the differences between background $\mathcal {\bar A}$ and focal sequence *x*. To be considered a well-conserved site, we require that a majority of the sequences in $\mathcal {\bar A}$ conform to the consensus sequence $\bar z$. As a default, we apply the majority rule and require 60% of the sequences to agree with the consensus. This threshold can be changed by the user.

Given both *x* and ${\bar z}$, we determine the set of sites with differences between the consensus and the focal sequence and denote this set of sites by $S_{\bar z\to x}$. For this purpose we consider gap characters like regular characters, i.e., $S_{\bar z\to x}$ also contains insertions and deletions in *x* relative to the consensus $\bar z$. We denote by $\bar z_{i}$ the sequence that is equal to $\bar z$ except at the single variable site *i*, where it matches *x*. Substitutions and indels are scored separately.

An insertion or deletion is treated as a single event independently of its length *ℓ*. The decision to handle gaps as a single unit was first based on the assumption that a unique evolutionary event is more likely to have caused the indel than two or more events acting on the exact same region. We tested this assumption, by measuring the structural impact of deletions of different lengths in biological RNAs. We found that the length of the deletion did not matter for the impact, but rather its location, specifically if it overlaps a paired region or not (more information in the Additional file 1). This is a consequence of the Turner model [67]: the energy penalty for the different loop types (hairpin, bulge, and interior loops) on slowly changes with the loop length, amounting to only about 1–3 kcal/mol between loop sizes of 3–30 nt.

Compensatory substitutions are those that leave the secondary structure unchanged by replacing one type of base pair (GC, AU, or GU) by another one that differs in one or both paired sites (e.g. AU →GC, or AU →GU). The SSS-test by construction considers single sites separately. Therefore we remove all sites that form compensatory substitutions. In order to identify these, we first compute the consensus structure of $\mathcal {\bar A}$ using RNAalifold [68] and the structure of sequence *x* with RNAfold (both tools from the ViennaRNA package [69]). We then compare both structures and consider a substitution or pair of substitutions as compensatory if they form a base pair both in the focal MFE structure of *x* and the consensus MFE structure of the background alignment $\mathcal {\bar A}$. All compensatory sites are removed from $S_{\bar z\to x}$.

All single nucleotide substitutions remaining in $S_{\bar z\to x}$ are scored using RNAsnp [65]. In a nutshell, RNAsnp quantifies the magnitude of structural change in response to a substitution relative to the expected change of secondary structure. The expectation is computed from the same base exchange in random sequences with the same length and GC content. For a given SNP, RNAsnp then returns a *p*-values for the hypothesis that the structural change caused by the SNP is larger than expected. Small RNAsnp*p*-values therefore indicate unexpectedly large structural changes in the structure of interest. RNAsnp is conceptually similar to several other tools to evaluate variation of RNA secondary structure, e.g. corRna [70], RNAmute [71], RDMAS [72], or SNPfold [73]. We employed RNAsnp both for its computational efficiency and several features that make its underlying model more realistic. The tool evaluates the Boltzmann ensemble of secondary structures rather than only the minimum energy structure, which provides more accurate information on the structural changes [65]. Instead of using arbitrary sequence windows or simply the global fold of the entire RNA, RNAsnp identifies the region of maximal structural discrepancy and evaluates the changes for this region. This at least approximates the fact that the structural impact of SNPs is expected to be localized e.g. due to proteins bound to a lncRNA.

Since each variation is scored independently, *p*-values are corrected for multiple testing using the Benjamini-Hochberg [74] procedure (with the more conservative Bonferroni method [75] available as well). The Benjamini-Hochberg procedure performs well with a larger number of *p*-values, which individually are ≥0.05, as happens quite often in our case with RNAsnp-based *p*-values. For the correction let *p*=*p*_1_≥*p*_2_≥⋯≥*p*_*n*_ be the collection of *p*-values. We then update the corrected set of *p*-values $\widetilde {p}$ using:

$$\begin{array}{*{20}l} \widetilde{p}_{1} & = \min \; \left\{ 1, \; p_{1} \right\}\\ \widetilde{p}_{i} & = \min \; \left\{ 1, \; \widetilde{p}_{i-1}, \; \frac{n}{(n-i+1)} p_{i} \; \right\} \end{array} $$

We then use the $\widetilde {p}$ to produce the substitution score

1 $$ s(x) = - \sum\limits_{i} \log \widetilde{p}_{i} \;.  $$

measuring the impact of the observed substitution in the focal sequence *x* relative to the expected changes of the secondary structure.

The RNAsnp tool cannot be used for insertions and deletions since its internal model for evaluating *p*-values is not designed for this type of variations. We therefore developed a separate model to score indels: for an indel of length *ℓ* we construct all sequences *z*_*j*_ that carry the indel after position *j* of the consensus. Since *z*_*j*_ and $\bar z$ differ in length, they cannot have the same structure. We therefore compute a modified reference structure *ψ*_*j*_ by constraining *z*_*j*_ to contain all base pairs of the consensus sequence $\bar z$ that are not affected by the indel. To this end we use the option of the ViennaRNA package to fold RNA sequences with user-defined constraints [76]. For comparison we compute the fold *ϕ*_*j*_ of *z*_*j*_ without constraints. To determine the structural impact of the indel we compute the structural difference *δ*(*ϕ*_*j*_,*ψ*_*j*_) of *ϕ*_*j*_ and *ψ*_*j*_ using RNAforester [77].

We then use a combination of rank statistics and relative structural impact to determine a *p*-value for the structural impact of indel *j*: let *r*(*j*) be the rank of indel *j* w.r.t. the size of its structural impact in decreasing order. Then *p*_rank_=*r*(*j*)/*n*, where *n* is the number of possible indel ranks. In addition we score the relative structural impact by *p*_struc_=(4*l*−*δ*(*ϕ*_*j*_,*ψ*_*j*_))/4*l*, with *l* the length of the sequence and *p*_struc_ clamped to 1/4*l* for extreme *δ*(·,·) contributions. The complete indel *p*-values (*p*=*p*_rank_+*p*_struc_) are aggregated as described above and yield a corresponding indel score contribution *s*^′^(*x*).

Finally, substitution (*s*(*x*)) and indel (*s*^′^(*x*)) scores are added to yield the final SSS-score score for the focal sequence *x*, using:

2 $$  \text{\texttt{SSS}-score}(x) = 2 s(x) + s'(x)  $$

As discussed in the previous section, both the assessment of nucleotide changes, and the evaluation of insertions and deletions has heuristic elements. Nevertheless, the nucleotide change model is grounded in a well-established model. Both *s*(*x*) and *s*^′^(*x*) are scores that convey information on how unexpectedly large the effect of the observed variations is on the secondary structures. They do not lend themselves to a direct interpretation e.g. as probabilities w.r.t. to a particular probabilistic model. Future versions of the SSS-test thus may well use an improved scoring model for either contribution. Similarly, the weighting factor of 2 in Eq. 2 was empirically determined to improve over equal weights. The scores SSS-score(*x*) thus serve as test statistics for which relevant cutoffs have to be determined empirically, since a concise statistical model for them is not available.

Manual analysis of families with different scores ranging from 0.0 to 30.0 showed that SSS-score≥10.0 is a suitable threshold determining that an element is under positive selection. For additional details we refer to the Additional file 1. The choice of the weighting factor in Eq. 2 as well as the threshold for SSS-score and the threshold of the family divergence, may vary for different applications. The values observed in this work are valid for primates, i.e., a set of phylogenetically very closely related taxa. For different projects, with more distant or more closely related species, the threshold may need to be adapted to best fit the data. In addition, the candidates should be subjected to functional testing for confirmation of the predictions.

### Implementation

The computation of selection scores is implemented in an automated pipeline using Perl and bash scripts (see pseudocode). In our implementation, the test statistic SSS-score is computed for all focal sequences $x\in \mathcal {A}$. If the input sequences are not aligned, muscle [78] is used to generate the necessary alignment. Additionally, species distance scores, *d*_*s*_, are computed for each sequence of the alignment to indicate the structural distance of the species to the consensus. The median species distance score is the family divergence score, *d*, which indicates the family’s structural uniformity (more details in the Additional file 1). However, as for the threshold on the selection score, the user should decide on a meaningful cutoff for the investigated data set.


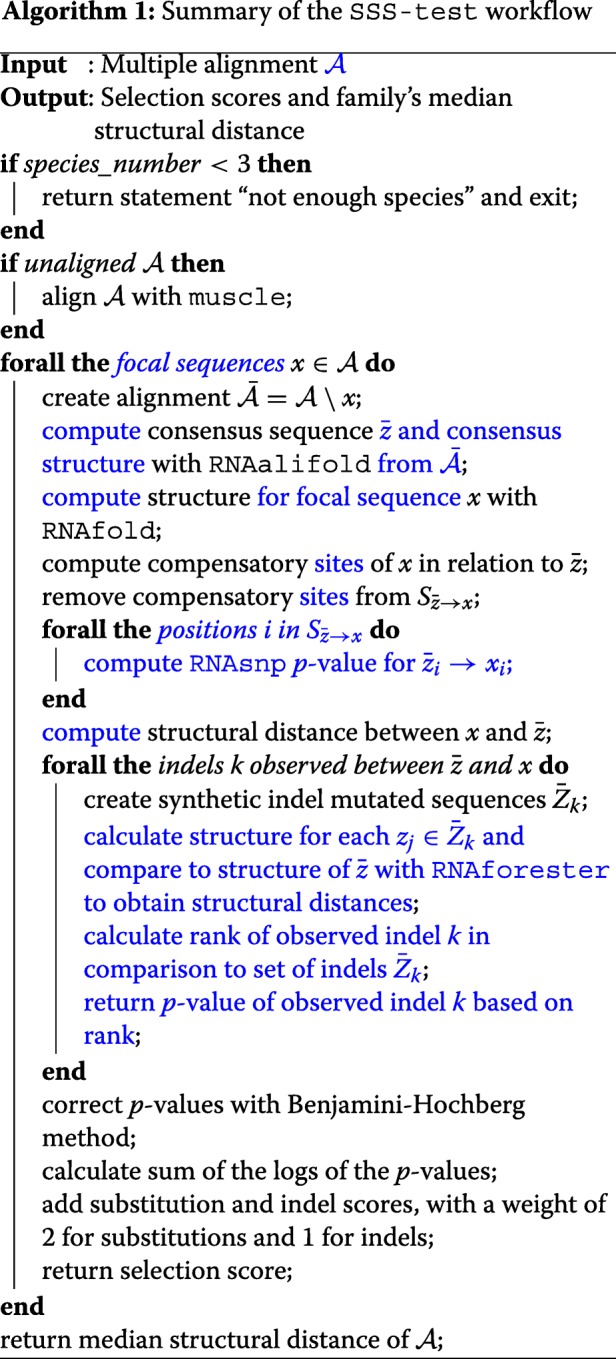


### Alternative approaches

We have also considered alternative ways to score the structural variations. The simplest model classified the substitutions into disruptive and non-disruptive sites based on their classification by RNAsnp. Based on this classification, an equivalent of the *K*_*a*_/*K*_*s*_ test becomes applicable (more information on the Additional file 1). Usually the number of sites is small so that the power of the test is low, however. In addition, false positive results were often seen in manually checked families, for structures that were extremely similar to their orthologs.

This inconsistency most likely came from categorizing sites only into two categories, either disruptive or non-disruptive, which is very difficult to do for ncRNA structures due to their biochemical properties. As an improvement we also built a statistical model based on a Poisson distribution of the counts of synonymous and non-synonymous sites instead of directly comparing the substitution counts. Although more robust, inconsistencies remained, most likely due to the same problem of using only a binary categorization of sites into either synonymous or non-synonymous. This led to the conclusion that an equivalent of the *K*_*a*_/*K*_*s*_ test is not appropriate for ncRNAs.

### Family divergence

We were most interested in identifying RNA structures that were conserved over a long period of time but showed lineage specific changes. Considering only structures with such conservation ensures to a large degree that the structure is biologically relevant. The SSS-test thus measures the structural divergence within a family of othologs, denoted as *d*.

Given the alignment $\mathcal {A}$ of a set of species, we denote by *A*_*s*_ the basepair probability matrix for the aligned sequence $s \in \mathcal {A}$, and by *B* the basepair probability matrix of the alignment $\mathcal {\bar A}$ itself. Furthermore, *P*_*s*_ is the set of base pairs in *s*, while *Q* is the set of base pairs in the consensus. Then we can calculate the derived sets *W*_*s*_=*P*_*s*_∩*Q* of shared base pairs, *X*_*s*_=*P*_*s*_∖*Q* of unique base pairs, and *Y*_*s*_=*Q*∖*P*_*s*_ of absent base pairs for each sequence *s*.

Using these, we can now calculate the divergence of each sequence *s* compared to its family:

3 $$ \begin{aligned} d_{s} &= \frac{100}{\text{length}(\mathcal{A})} \\ &\quad {\times}\left(\sum_{ij \in W_{s}} \left| A_{s,ij} - B_{s,ij} \right| + \sum_{ij \in X_{s}} A_{s,ij} + \sum_{ij \in Y_{s}} B_{s,ij} \right) \end{aligned}  $$

We then calculate the family divergence as the median over the individual divergence scores *d*=median_*s*_ *d*_*s*_. Manual analysis of families with different *d* scores (ranging from 0.0 to 65.0) revealed that (*d*≤10.0) is a suitable threshold indicating low family divergence (additional details are provided in the Additional file 1).

## Results

### Benchmarking

#### Biological controls: SSS-scores indicating negative selection for small ncRNAs and positive selection for human HAR1

As a plausibility check for the SSS-test we used collections of small ncRNAs, which are known to be structurally conserved [14, 61]: miRNAs, snoRNAs, and tRNAs (family conservation overview can be seen in Additional file 1: Figures S8 and S9). These collections of ncRNAs were expected to receive low selection scores, indicating negative selection. As expected, all three groups showed strong evidence for negative selection, while pseudo-tRNAs exhibit the least constraint (Additional file 1: Figure S10).

Despite the lack of direct experimental evidence for HAR1 functioning based on its structure, we also applied our test to HAR1 as the only, at least putative example of a positively selected structure. We detected a signal for positive selection that is exclusive to the human HAR1 structure (*s*=12.8), while all other seven primate species in the input set displayed strong negative selection signals (*s*=0.0). This is in agreement with [55].

#### Synthetic data sets: SSS-test can distinguish between negative and positive selection models

In addition, we also produced synthetic data sets in order to evaluate whether our SSS-test test can distinguish between different degrees of divergence and between positive and negative selection.

One optimization function simulated negative selection, in which changes were kept if they maintained the ancestral structure. Another simulated loss of selection pressure (random evolution), in which every change was kept. The third one simulated positive selection, in which the origin was a Y-shape and the changes were kept if they caused a change towards a cloverleaf structure.

**Fig. 1:**
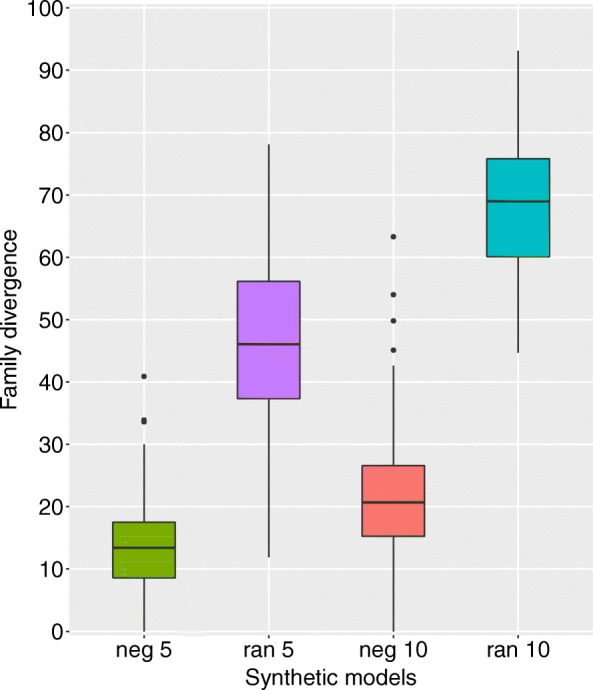
Structural divergence *d* of synthetic data sets for families evolved under simulated negative selection pressure (neg) compared to unconstrained (ran) evolution. Each data set is composed of 100 families, evolved from one ancestral sequence to five extant sequences, differing by 5 (left) or 10 (right) accepted substitutions from the ancestor

We found that the divergence of the ortholog families under negative selection was distinctly lower than the divergence of families in the random evolution set (Fig. 1). This shows that the SSS-test can correctly distinguish between constrained and highly diverged families.

**Fig. 2:**
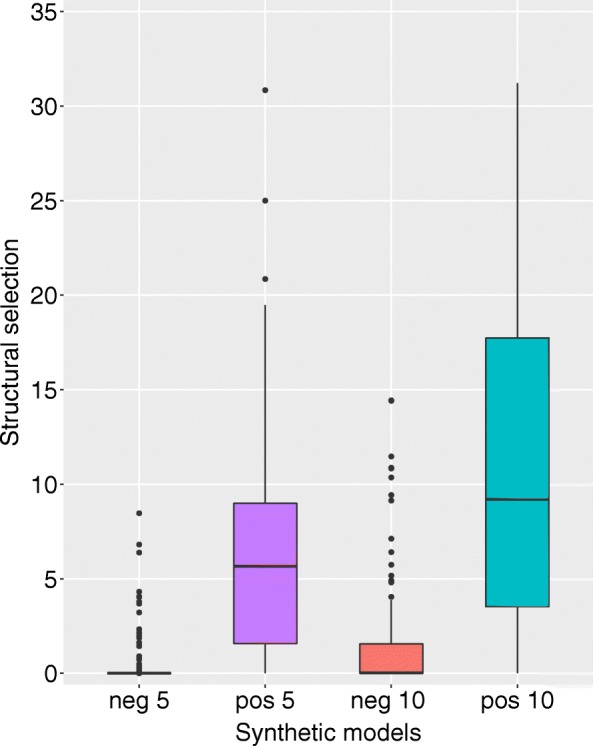
SSS-score *s* of synthetic data sets with simulated negative evolutionary constraints compared to simulated positive selection. Each data set is composed of 100 sequences, evolved from one ancestral sequence to one extant sequence, differing by 5 (left) or 10 (right) accepted substitutions from the ancestor

We can also detect a clear difference between the set of families that evolved under negative selection pressure in comparison with the ones that evolved under positive selection pressure, with the families under positive selection having higher scores (Fig 2).

### Local structures of lncRNAs are mainly negatively selected

To showcase our SSS-test, we conducted a survey of positive selection on structural elements of human lncRNAs. We first computed local structural blocks (conserved local structures shared between at least three species) and then applied the SSS-test on them. For the 15 443 primate lncRNA ortholog families reported in [20], a total of 10 396 blocks were calculated, with orthologs in at least three species and with *d*≤10.0 (Additional file 1: Table S3).

For this collection of lncRNAs 87 613 local blocks were calculated initially (Additional file 1: Table S3). On average, this amounts to 5.7 blocks per lncRNA family. 77 217 of these blocks have orthologs in only one or two species and/or have a *d*>10.0 and thus are excluded from the analysis. After this filtering step, we retained 0.7 blocks per lncRNA family with at least three species and *d*≤10.0. Since we do not expect local secondary structures to be functionally important for all RNAs, the relatively small size of the remaining set is not at all unexpected. We note, furthermore, that the data set relies on coordinates and orthology assignments from ref. [20], so that a non-negligible part of the excluded blocks is due to the quality of the primate genome assemblies and alignments available at the time. For the retained blocks, we observed a substantial level of conservation among these local structures, comparable to the small RNA databases (Additional file 1: Figures S8, S9 and S10). This confirms previous reports that lncRNAs as a group are under negative selection [10, 15, 21, 22] and that conserved RNA structures are common throughout the non-protein-coding parts of the genome [43–45, 79].

The results described in this section were obtained by applying the SSS-test with the default threshold of at least 60% of the sequences agreeing with the consensus for a site to be considered well-conserved (for details we refer to the Theory section). For this primate dataset, 98.6% of the sites are well-conserved. By changing the threshold from 0 to 100%, the proportion of well-conserved sites changes only slightly: from 100% to 87.7% of well-conserved sites (Additional file 1: Figure S16). This high value is expected due to the close phylogenetic distances among the primates.

Using the default parameters of the SSS-test, we observed that 0.77% of the sites changed human specifically. 0.74%, 0.98%, 1.57%, and 3.80% of the sites changed compared to the consensus sequence specifically in pan, gorilla, orangutan, and rhesus macaque, respectively (Additional file 1: Table S5). The number of species-specific substitutions varies slightly if the threshold for well-conserved site is changed, (see Additional file 1: Figure S17 for details), however, the overall pattern of more species specific sites with higher evolutionary distance from humans stays the same. In addition, compensatory sites account for 7-10% of the variation among the five primates (Additional file 1: Table S6).

### Positively selected RNA structures in human lncRNAs

In order to identify candidates for lineage-specific selection we considered only the subset of 10 396 local blocks with an overall low structural divergence, i.e., a likely well-conserved ancestral structure. Using a stringent selection score cutoff of *s*≥10.0 (see Methods for details) we detected 1390 local structures that show signs of positive selection. More than half of these structures (738) show significant differences between the rhesus macaque and the Great Ape lineage (in 716 distinct lncRNAs) (Table 1). Among the Great Apes, we identified in the orangutan lineage 315 local structures with high SSS-score in 312 distinct lncRNAs. In the gorilla lineage we found 136 structures with signs of positive selection in 135 distinct lncRNAs. In *Pan* (chimpazees and bonobos) 90 structures were found as potential positively selected candidated in 89 lncRNAs. High selection scores were detected in 111 local structures of 110 human lncRNAs. The one human lncRNA with two distinct structures under potential positive selection is ENSG00000246548 (LINC02288). The numbers of candidates under positive selection seems roughly proportional to the evolutionary distance between species, which is not unexpected. (Table 1).

**Table 1:** Characterization of local structural selection of lncRNAs

Species	Local structures	Conserved (*s*≤2)	Positive (*s*≥10)	
Human	8934	8179 (91.6%)	111	(1.2%)
Pan	8736	7997 (91.5%)	90	(1.0%)
Gorilla	8080	7199 (89.1%)	136	(1.7%)
Orangutan	6435	4802 (74.6%)	315	(4.9%)
Macaque	5113	2659 (52.0%)	738	(14.4%)

Only the low diverged set was considered in this analysis. Percentages of conserved and positive structures are relative to each species’ number of representatives

In order to estimate the FDR of the SSS-test survery, we used the 8934 alignments of local structures containing a human sequence and shuffled them with SISSIz [42] as described in the Methods section. Using the same cutoffs for *d*_*s*_ and SSS−*score* as for the real data, we obtained 50 predictions, amounting to an FDR of 45%. A closer inspection shows that the shuffling does not completely destroy the signal for positive selection in the real data: repeated shuffling of the positive predictions in the real data shows that about 18.5% of these shuffled alignments still yield a positive result with the SSS-test. Hence about 20 of the 50 predictions in the shuffled sets correspond to the predictions on the real data, reducing the estimated FDR to less then 30%. This is comparable to the FDR of most of the surveys for negative selection: For instance RNAz 2.0 reported 54% for a human survey [80], a FOLDALIGN-bases survey on the ENCODE regions obtained about 50% [43]. A hybrid of SISSIz and RNAz achieves 5–22% [44], and a recent cmfinder-based screen with score cutoffs depending on GC content estimates a FDR of 14±5*%* [45].

We separately investigated SRA, Xist, and HOTAIR for signs of positive selection in humans within the primate group. No signal of positive selection was detected for these three well-studied lncRNAs.

### Profile of positively selected structures of human lncRNAs

**Fig. 3:**
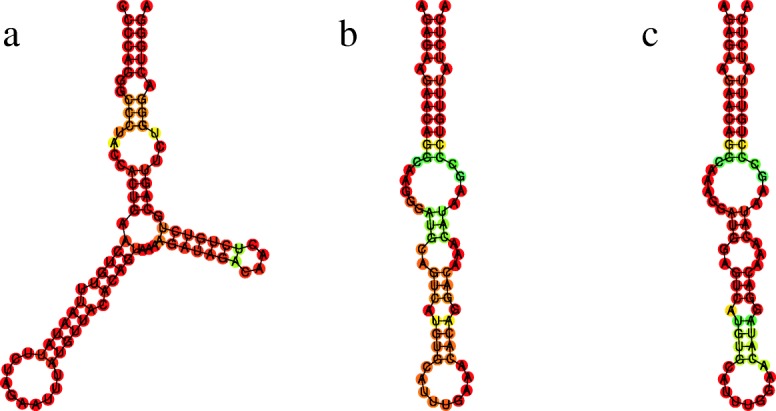
Local lncRNA structure LINC02217sub5: **a** human, **b** pan and **c** gorilla; Only the human structure obtained an SSS-score indicating positive selection with *s*=16.2, while the data indicates strong negative selection for the other species (*s*=0.0). Structures are represented by their minimum free energy. Base colors are assigned according to their pairing frequency in the structure’s ensemble. Shades of red occur in ≥90*%* of the ensemble, shades of green/yellow denote increasing probabilities from ≥50*%*. For unpaired bases, shades of red denote increasing unpairedness

**Fig. 4:**
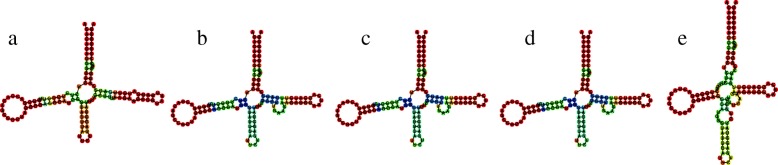
Local lncRNA structure SIX3-AS1sub11: **a** human, **b** pan, **c** orangutan, **d** gorilla and **e** macaque. Only the human structure obtained an SSS-score indicating positive selection with *s*=12.2 while the other species have strong negative selection scores (*s*=0.0). Structures are represented by their minimum free energy. Base colors are assigned according to their pairing frequency in the structure’s ensemble. Shades of red occur in ≥90*%* of the ensemble, shades of green/yellow denote increasing probabilities from ≥50*%*. For unpaired bases, shades of red denote increasing unpairedness

We next investigated how the local structures with signs of positive selection have been altered. Interestingly, we detected changes in the form (exemplified in Fig. 3) as well as changes in the stability (Fig. 4) of the structures. For instance, local structure 11 of *SIX3-AS1* shows little difference in the minimum free energy structure, but has considerably gained in stability in humans, as shown by the increase of the base pair stability in all three inner stems (Fig. 4). Increase in stability could for instance fine-tune interactions, having an important impact in function, as is the case of the human HAR1, which has acquired higher stability in the human lineage [58].

In our initial analysis we had orthologs of SIX3-AS1 only in human, pan, and orangutan. To identify the orthologs in gorilla and rhesus macaque (Additional file 1: Figure S11), we performed genome-wide scans using Infernal v1.1.1 [32]. First, a covariance model was built and calibrated for the sequences of human, pan and orangutan and the consensus structure yielded by RNAalifold [69]. After building the covariance model, we searched for homologous structures in gorilla and macaque, obtaining very likely hits (score of 155.1 and an e-value of 1.5×10^−31^ for gorilla and score of 150.7 and an e-value of 1.7×10^−30^ for macaque).

The macaque structure is located in one of the exons of the lncRNA and the gorilla structure is located close but not inside the annotated locus (Additional file 1: Figure S11). The two recovered structures show a similar structural pattern as pan and orangutan, being also less stable than the human structure (Fig. 4). When including these two sequences into the family and recalculating the SSS-scores, the signals are maintained (human SSS-score *s*=12.2 and the other four species *s*=0.0). This corroborates our predictions that the human structure is under positive selection when compared to closely related species, and has acquired higher stability during evolution.

In contrast to SIX3-AS1, there is still little or no functional annotation for most candidate lncRNAs. Only 49 of the 110 lncRNAs have an ENSEMBL Gene ID and only 20 of them have been associated an HGNC gene symbol yet (Table 2). To gain more information about the 110 lncRNAs that have positively selected structures in the human lineage, we analyzed in which tissues they are expressed. Based on the expression data reported in [20] we found that six of the lncRNAs are expressed in all nine reported tissues (brain, including developing brain, cerebellum, liver, heart, kidney, placenta, ovary, testis and stem cells), eight have no detected expression in humans and 16 are expressed only in one tissue in humans.

**Table 2:** Human lncRNA candidates with signs of positive selection in local structures

Gene name	Transcription age	Sequence age	Nb species transcribed	Nb species with sequence	ENSEMBL gene ID
RRS1-AS1	African apes	Great apes	3	4	ENSG00000246145
LINC01939	African apes	Great apes	3	4	ENSG00000228799
LINC01839	Primates	Primates	4	4	ENSG00000227509
LINC01802	Primates	Primates	5	5	ENSG00000225064
LINC01724	Primates	Primates	5	5	ENSG00000227421
LINC01693	Primates	Primates	5	5	ENSG00000227764
MACC1-AS1	Primates	Primates	5	5	ENSG00000228598
TRPM2-AS	Primates	Primates	5	5	ENSG00000230061
LINC01258	Primates	Primates	5	5	ENSG00000249534
PLUT1	Primates	Primates	5	5	ENSG00000247381
LINC01345	Primates	Primates	5	5	ENSG00000226374
MDC1-AS1	Primates	Eutherians	2	6	ENSG00000224328
LINC01790	Therians	Therians	3	7	ENSG00000230173
LINC02042	Eutherians	Eutherians	5	5	ENSG00000240893
LINC02092	Eutherians	Eutherians	5	6	ENSG00000234721
LINC01738	Eutherians	Eutherians	6	6	ENSG00000227947
LINC02288	Eutherians	Eutherians	6	6	ENSG00000246548
LINC02217	Eutherians	Eutherians	6	6	ENSG00000248455
DNMBP-AS1	Mammals	Amniotes	6	9	ENSG00000227695
SIX3-AS1	Tetrapods	Tetrapods	9	9	ENSG00000236502

The evolutionary age and expression information was taken from [20]. Gene names were retrieved from the ENSEMBL database. Only transcripts that have been assigned an HGNC ID are shown

Interestingly, the positively selected lncRNAs tend to be expressed in more tissues than lncRNAs in general (Spearman’s rank correlation: *ρ*=0.78, p =0.0081, Additional file 1: Figure S12). While 2% of the lncRNAs expressed in 8 tissues are under positive selection, this is the case for less than 0.5% of those expressed in a single tissue or not expressed at all in the data set from [20]. Just as a characterization of this data set, we plotted the distribution of number of exons, and saw that most lncRNAs have only one or two exons (Additional file 1: Figure S13).

### Evolution of lncRNAs associated with psychiatric disorders

Given that HAR1’s expression is dysregulated in Huntington’s disease [81, 82] and that two of the lncRNAs with potentially positively selected structures on the human lineage (SIX3-AS1 and TRPM2-AS) are antisense to genes involved in brain disorders [83, 84], we decided to have a closer look at lncRNAs that have been reported to be related to psychiatric disorders (PDs). Positive selection in such lncRNAs might indicate functional changes related to human brain evolution.

We collected 26 lncRNAs from public databases and the literature that have been reported to be involved in PDs (Suppl. Tab. 4). These 26 lncRNAs contain 362 local blocks consisting of 1331 local structures. 942 of those had a low family divergence (*d*≤10.0). From those, 32 have a positive selection score (*s*≥10.0), with three of them in the human lineage (Table 3). Considering the small number of analysed families, secondary structures could be analysed by manual inspection. We saw that if the threshold for considering positive selection is relaxed in this data set from *s*≥10.0 to *s*≥4.5, another 11 local structures in humans can also be considered as candidates for having evolved under positive selection (Table 3).

**Table 3:** ENSEMBL IDs and SSS-scores of local structures with signs of a positive selection/weak positive selection in humans

lncRNA family (*block ID*)	Selection score
**MIATsub92**	**21.2**
**NEAT1sub2**	**14.5**
**LINC00689sub32**	**11.0**
LINC00689sub40	8.9
LINC00689sub38	7.4
MEG3sub15	7.0
H19sub7	6.8
SOX2-OTsub27	5.9
MEG3sub1	5.4
BDNF-ASsub18	5.0
LINC02151sub5	4.9
MIATsub86	4.7
MIATsub31	4.6
NEAT1sub120	4.6

Marked in bold are local structures with SSS-score above 10.0

**Fig. 5:**
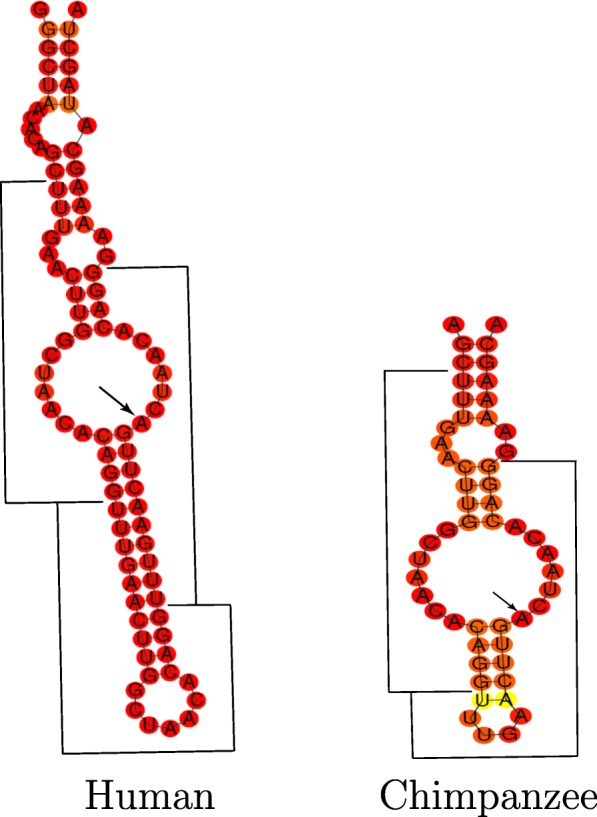
Minimum Free Energy (MFE) structures of MIATsub92 local structures of Human and Pan. The duplication of a TTTGAACTTGGCTAACACAGG sequence in the human lineage might have driven the evolution of the structure towards a more stable structure. Prevailing red regions exhibit well-defined structures with probabilities close to 1 for paired and unpaired bases. Duplicated regions are labeled with horizontal and vertical lines, and G/A nucleotide substitution is marked with an arrow. Bonobo has the same sequence as the chimpanzee

Among the positively selected human structures, MIATsub92 shows the highest selection score (SSS-score = 21.2). The human structure and its ortholog in chimpanzees contains a tandem duplication of the sequence TTTGAACTTGGCTAACACAGG (Fig. 5), with a substition of a G to A in one of the duplicates (TTTGAACTTG(G/A)CTAACACAGG). Unlike the chimpanzee ortholog, the human structure contains another duplication of the TTTGAACTTGGCTAACACAGG sequence. It seems that this duplication has had an essential impact on the human MIATsub92 structure and might have contributed also to an increase in stability of the human structure compared to its counterparts. It is worth noting that TTTGAACTTGGCTAACACAGG also has a deletion variant in humans with highest population frequency of 0.36. This means that some human individuals still possess the ancestral version of MIATsub92, indicating that this human specific change likely occurred very recently and is not yet fixed. Although this deletion variant is not reported to have a phenotypic effect, it could lead to destabilization of structure, as denoted by decreasing base pair probabilities and shortening of the lower stem, as seen in the chimpanzee ortholog (Fig. 5).

Apart from MIATsub92, two other local structures in MIAT (MIATsub31 and MIATsub86) also show high structural differences in the human lineage in comparison to non-human primates. Greater structural stability of MIATsub31 in humans is noticeable when comparing the centroid structures (Additional file 1: Figures S14 and S15). MIATsub31 contains UACUAAC repeats, which are known to be important for binding of splicing factors [85, 86]. Importantly, in both, humans and non-human primates, these repeats are always found within unpaired regions, and the red coloring of the nucleotides in these internal loops indicates high probability of unpairedness. It seems as if selection was driving the evolution of MIATsub31 towards higher stability in humans, while at the same time it was important that UACUAAC remained unpaired. This suggests that the binding specificity of splicing factors is dependent on internal loops, and not simply a sequence of repeats.

## Discussion

We have introduced here a statistical test for positive selection on RNA secondary structure. It focuses on orthologous structures that are otherwise conserved across taxa, thereby identifying candidates with species-specific functional changes in ncRNAs. The SSS-test is implemented as an easy to use command line tool, that we expect to be relevant for a number of future genomic and evolutionary studies. It requires as input a simple FASTA file, and can be used for any type of ncRNA, including the yet largely uncharacterised group of lncRNAs.

While the SSS-test was designed primarily to detect positive selection, it can also be used to identify ncRNAs under negative selection. Since genes with very small scores are candidates for evolving under negative selection, the SSS-test may be employed to complement other available methods for assessing structural conservation. In addition, orthologous groups of local structures with high divergence scores within the family might indicate genes that evolve under relaxed selective constraints. For instance, lncRNAs that contain only local structures with high family divergence are probably transcripts for which the secondary structure is not functionally relevant.

In the study reported here we required at least three orthologs for the analysis. Depending on the system under consideration, a sufficient number of closely related species may not always be available, however. In this case, one could consider a pairwise version of the SSS-test. While this could easily be implemented, the interpretation of the results will necessarily be quite different: In a pairwise setting, it is unknown which sequence represents the ancestral state, hence one can only test whether the structures are unexpectedly different. While positive selection in one of the lineages is possible, divergent evolution should also be considered.

The current version of the SSS-test uses the relatively simple SSS−*score* as decision variable. Although it is based on established measures of structural variability and it is empirically capable of distinguishing modes of selection, it does not derive from a stochastic model of the evolution of RNA secondary atructure. In particular, the way in which in/dels are evaluated is rather ad hoc. It will be of interest for future work to find a parameter with a better theoretical foundation as a replacement for the SSS−*score*. This will likely imply that the covariation of paired nucleotides will have to be taken into consideration as well.

Gene duplications are often – but not necessarily – accompanied by positive selection, leading to functional changes in one or both duplicates [87, 88], see e.g. [89] for a case study. In this context, it is imperative to accurately distinguish between (co)orthologs and paralogs. As any statistical test for positive selection, also our test could report false positives when inadvertently including paralogs. This is a general concern in the analysis of protein-coding genes and also pertains to many ncRNAs including miRNAs, snoRNAs, and tRNAs that are frequently duplicated [90–92]. On the other hand, the pairwise version of our SSS test mentioned above could also be applied to a pair of duplicated ncRNAs to assess whether they might evolve under positive selection. In our case study of local structures of lncRNAs in primates, we found an extra duplication in the human structure of MIATsub92, which increases the stability of the local structure and elongates its lower stem, confering it a signal for positive selection (Fig. 5). This example suggests that tandem duplications could greatly impact the evolutionary trajectories of lncRNAs, and should therefore be considered in further studies of ncRNA selection. The contribution of segmental duplications to lncRNAs appears to be much smaller than for the families of small RNAs [93], despite some exceptional cases such as FAM230 [94]. Short local duplications may cause alignment problems that may translate into erroneous results of the SSS-test. Thus we strongly recommend to manually inspect sequence alignments passed to the SSS-test.

We applied our test to an evolutionary analysis of primate lncRNAs, identifying 111 local structures with signs of positive selection on the human lineage. These comprise 110 lncRNA genes, with one of those containing two local structures with signs of positive selection. Most of the candidates are unknown, and those with some description are: PDX1 associated lncRNA (PLUT1) and another six candidates that overlap proteins that are antisense to them (RRS1, MACC1, TRPM2, SIX3, DNMBP and MDC1). The power of the test is inherently limited by the amount of sequence variation among the lineages considered. Most likely, therefore, we experience a large false negative rate due to small divergence between primate genomes. Given that the power of the test is limited by the moderate sequence divergence between primates, the lncRNAs identified in this study are probably only the proverbial tip of the iceberg.

Of the proteins mentioned above, two have known important functions in the brain: TRPM2 (Transient receptor potential melastatin 2) is an ion channel expressed in the brain. It is essential for cell survival by modulating mitochondrial responses and has also been associated with neuroblastoma [83]; The SIX3 protein is a transcriptional regulator that plays a role in eye development and is associated with cephalic disorder [84]. The SIX3-AS1 lncRNA (also known as SIX3OS) modulates the SIX3 protein by acting in *trans* to regulate retinal development. SIX3-AS1 has an essential role in regulating retinal cell specification. Although it is directly antisense to SIX3, it does not regulate its expression, but is rather acting as a molecular scaffold directly binding to histone modification enzymes directing them to SIX3 target genes [95]. The specifics on the SIX3-AS1 interaction with its partner proteins still remain unknown, but it seems worthwhile to study the role of this positively selected structure in such interactions, and whether the increased stability in humans changes or fine-tunes interactions, when compared to other primates.

The proteins antisense to the other candidates have varying functions outside of the brain. MACC1 is an immunogene [96]. PDX1 is a regulator of pancreas development and *β* cell differentiation and its antisense lncRNA, PLUT1, is potentially associated with diabetes and affects chromatic structure and the transcription of PDX1 [97]. RRS1 is involved in ribosome biogenesis [98]. MDC1 has a role in cell cycle and cancer control [99, 100]. And the function of C5orf66 has not been characterised yet.

While functional annotation of most other candidate lncRNAs is still pending, it is interesting to note that there is a tendency for lncRNA that are expressed in many tissues to show more frequently signs of positive selection than lncRNAs that are expressed in only a few tissues. The broader tissue expression suggests that structural changes might often have a ubiquitous effect instead of a very localized one restricted to a few tissues.

LncRNAs have been strongly associated with brain development, synaptic plasticity, neural functioning as well as neurodegenerative and psychiatric disorders [101–104]. Human evolution has been characterized by an incease in brain size and complexity, followed by an improvement of cognitive abilities. Notably, dysfunctions of cognitive skills are observed in psychiatric patients [105]. There might be a causal link between human brain evolution and increased susceptability to PDs, as those are mostly human specific disorders. Candidates such as HAR1 and SIX-AS1 prompted us to investigate if also other lncRNAs associated with PDs have been positively selected in humans.

We detected at least three lncRNAs with strong signs of positive selection on the human lineage, MIAT, NEAT, and LINC00689. Several lines of evidence demonstrate an important role of MIAT RNA in the development of schizophrenia [85, 106, 107] and in substance dependence, as its expression is upregulated in the nucleus accumbens of cocaine and heroin abusers [108, 109]. In addition, aberrant expression of MIAT is observed in neurovascular dysfunction contributing to the pathogenesis of Alzheimer’s disease [110]. The strongest signal of positive selection in our data was in the local structure MIATsub92, which contains a human specific duplication that seems to be very recent and not yet fixed in the human population. This duplication has an effect on the shape and stability of the structure. MIAT RNA was shown to co-localize within a nuclear compartment that is enriched in splicing factors [111]. Its expression is down-regulated upon activation of neurons, which allows disassociation of splicing factors that further mediate splicing of targeted genes [85]. In post-mortem brains of schizophrenic patients, expression of MIAT is also down-regulated, and changes in expression level of MIAT result in dysregulation of alternative splicing [85]. MIATsub31, with weak signs of positive selection in humans, contains repeats that are important for the interaction of MIAT with the splicing machinery. These repeats have always been observed with high probability to be located within unpaired parts of the structure, implying importance of internal loops in recognition and binding of splicing factors. It is tempting to speculate that differences in splicing patterns between human and non-human primate brains are in part caused by evolutionary changes in MIAT.

## Conclusions

The SSS-test provides an efficient statistical approach to assess whether small ncRNAs or local structures of lncRNAs might evolve under positive selection. Our work thus complements previous studies on detecting negative selection of ncRNAs. The SSS-test evaluates whether ncRNA genes harbour an excess of evolutionary events, such as substitutions and/or indels, that lead to a rather large structural change. Therefore, it can also provide information on whether secondary structure is under negative selection, and whether relaxed constraints make a functional role of the RNA structure unlikely. An advantage of our test is that we consider the structure directly as the phenotype, instead of analyzing the sequence conservation to detect positive selection. One has to keep in mind, however, that RNA secondary structure prediction is not perfect and hence false positive predictions are unavoidable; additional experimental verification thus is strongly advised.

We demonstrated that the SSS-test is capable of detecting lineage-specific positive selection on secondary structures in genome-wide surveys. Given the limited power of the method, we suspect that the approximately one hundred candidates in the human lineage are a lower bound. In addition, the detection of lineage-specific positive selection in genes associated with cognitive disorders in humans lends further credibility to our method.

## Data and methods

### Evaluation of the SSS-test on small ncRNA databases

We applied the SSS-test to known examples of positive and negative selection. To the best of our knowledge the 118 nucleotide region of the *Human accelerated region 1* [55] is the only available control for positive selection on non-coding structures to date [58]. It is well established that HAR1 is stable in humans and differs from its orthologous structure in chimpanzee and other non-human primates [56, 57]. It is extremely conserved across vertebrates but has 18 fixed human changes [55], which stabilized its structure, likely caused by positive selection [58].

As negative controls for our test, we used three databases of structurally conserved small ncRNAs: (i) miRNAs (miRBase [33], release 21), (ii) CD and HACA box snoRNAs [90], and (iii) tRNAs (personal communication). We only selected sequences of the following primates from each database: human, chimpanzee, gorilla, orangutan, and rhesus macaque (with the exception of the snoRNA database which does not contain orangutan sequences). We analysed 167 microRNA families, 176 snoRNA families (containing CD and HACA box snoRNAs) and 511 tRNA families (containing functional tRNAs as well as pseudo tRNAs). Each family of these databases contained only one sequence per primate species, to avoid species bias. Only families with low-divergence (*d*≤10.0) were retained for further analysis, resulting in 142 microRNA families, 78 snoRNAs families and 141 tRNA families for our analysis of selection.

### Evaluation of the SSS-test on synthetic data sets

After evaluating the SSS-test using biological RNAs, we evaluated it using *in silico* designed sequences. In this way, it is possible to simulate evolution and keep a tighter control on the selective pressures and how the families are constructed. We designed two experiments. The first is designed to test if the SSS-test can differentiate between low and high divergence of individual families. The second experiment tests if the SSS-test can differentiate between negative and positive selection within low-diverged families.

To answer the first question, we simulated evolution from one origin or ancestral sequence to five extant branches. This provides us with five evolved sequences that compose one family, similar to the real biological data we worked with.

To answer the second question, we simulated evolution from one origin to one extant branch but kept the other four branches unchanged compared to the ancestral origin. This simulates a case in which the family is composed of four species that have kept the ancestral sequence (due to extreme negative selection) and one species that changed its sequence due to a different evolutionary pressure.

The synthetic data sets were created with RNAdesign [112], with each family starting from a randomly created RNA sequence of 150 nt. For each database, 100 families were generated and subjected to the SSS-test. To simulate evolutionary pressures, the starting sequence is randomly mutated, whereby a mutation is accepted or rejected according to the *different* optimization functions which we detail below. The simulation evolves the origin until *n* changes are accepted. We performed two simulations for each set, with *n*=5 and *n*=10.

We simulate the following cases:

- negative selection (*f*_neg_), as a pressure to maintain the original structure, where deviation from the ancestral secondary structure is penalized;
- random evolution (*f*_rand_), with no pressure towards any goal, with any mutation being accepted; and
- positive selection (*f*_pos_), where an ancestral Y-shaped structure experiences mutations and the optimization function provides pressure towards a cloverleaf structure.

Denote by *a* be the ancestral sequence and let *m* be the current sequence being designed by RNAdesign. Consider *ε* as a stabilizing parameter that keeps the energy of the evolved sequence at least at half of the original sequence to prevent degenerate structures from forming:

4 $$ \varepsilon(a,m) = \left(\max \ 0 \ \left(\text{mfe}(m) - \frac{\text{mfe}(a)}{2}\right)\right)  $$

As long as the sequence *m* has a minimum-free energy at least half of the ancestral sequence, *ε*(*a*,*m*) accepts the proposal *m*. Otherwise, the large penalty will make acceptance extremely improbable.

Similarly, we can constrain both the basepair and shape distance [113] of *a* and *m*. For the basepair distance, the penalty function reads as in Eq. 5, while the shape distance is based on a simplified alignment cost of the two shapes.

5 $$  \varDelta(a,m) = \text{base pair distance}(a,m)  $$

Shapes [113] are coarse-grained representations of secondary-structures. Each shape represents a wide range of sequences and their secondary structures that fold into the same “rough” structure. There are different levels of shape representations, ranging from 1 to 5, which indicate the abstraction level. We used the most abstract representation (level 5), which encompass a wide set of possible sequences that fold into the same abstract structure.

For case (i) of negative selection, we constrain the basepair distance of the centroids of the origin and extant sequence. Using a very large penalty for a basepair distance >0, we prevent structural divergence of the centroid. This penalty is given in addition to the energy penalty *ε* discussed above. This results in the following optimization function

6 $$ {f}_{\text{neg}}(a,m) = 1000 \left({\varDelta}_{\text{centroid}}(a,m) + \varepsilon(a,m) \right)  $$

In contrast, case (ii), random evolution, has no penalties at all, here the optimization function is constant 0, independent of the extant sequence:

7 $$ f_{\text{rand}}(a,m) = 0  $$

Finally, for case (iii), positive selection, we compute the RNA shapes (level 5) of the centroid of the mutating extant sequence. We penalize distance to a cloverleaf-shaped target (level 5). This simulates the pressure on the new structure, which is constrained to move from a Y-shaped origin towards a cloverleaf ([[][][]]) target:

8 $$ \begin{aligned} f_{\text{pos}}(a,m) = \text{gibbs}(m) &+50\varDelta_{\text{shape:5}}(\text{\texttt{[[][][]]}},m)\\ &+1000 \; \varepsilon(a,m) \end{aligned}  $$

It is important to notice that these experiments are intended to provide a control for the SSS-test and its ability to differentiate between differently constructed families. The intention is *not* to provide a full model of simulated evolution in a biological sense. The latter is a very difficult problem, and out of scope for this contribution.

### Structural selection of lncRNA local structures

To illustrate an interesting application of the test, we searched for lncRNA structures that are positively selected in human using a primate group which includes human, pan (including both chimpanzee and bonobo), gorilla, orangutan, and rhesus macaque. The data of [20] provides coordinates in BED format for 15 443 lncRNA families, including orthologs of these five primates. We used an in-house C-program to retrieve the sequence information from the genomic DNA data based on the coordinates provided. We used muscle to compute alignments of orthologous lncRNAs.

It has been observed that most base-pairing interactions in longer RNAs occur within a short span of 150-200 bp [114]. Taking this into consideration, it is also expected that evolution acts on these smaller modules of lncRNAs (local folds), rather than on the entire structure. Therefore, it is more reasonable to search for positive selection locally than globally in lncRNAs. Local structural elements were identified separately for each species using RNALfold, a component of the ViennaRNA package that computes minimum energy structures with restricted base pair span [115].

The most energetically stable local structures were chosen for each species in a way that all chosen structures can co-exist with each other (they do not overlap). Local structures from different species were considered orthologous if they overlap at the starting position with regard to the alignment. To allow for a little bit of freedom, the starting positions could diverge by at most 30% of the length of the sequences. Only regions containing orthologous structures from at least three species were considered, and these are defined as conserved *blocks*. In total we identified 19 408 blocks with at least three ortholog species. Of these 10 396 have low family divergence (*d*≤10.0) and were kept for complete selection analysis with the SSS-test.

Information on the evolutionary age and tissue-specific expression patterns were extracted from the supplemental files provided in [20]. This data also includes the number of species with orthologous sequences in the lncRNAs and detectable expression.

### Estimation of the false discovery rate

The false discovery rate (FDR) is defined as the expected fraction of false discoveries among all discoveries. It can be estimated for a given “foreground” data set by comparing the number *F* of positive test results in the “foreground” with the number *R* of positive test results a “background” data set of the same size. The latter is conveniently obtained by shuffling each of the “foreground” alignments using SISSIz -s [42]. Since this shuffling method destroys the correlation of alignment columns, and hence the secondary structure, we may consider all positive test results on the shuffled alignments as false positives. If this assumption is violated, and the shuffled set retains some of the foreground signal, we only obtain an upper bound, i.e., FDR=*R*/*F*.

Empirically we found that our shuffling procedure indeed does not completely remove the “foreground” signal. Using SISSIz -s to produce 20 independent randomizations of the “foreground” predictions, we estimate the fraction of tests *f* at which the signal is retained. Under these circumstances, we can refine the estimate of the FDR and use FDR=(1−*f*)*R*/*F*.

### LncRNAs involved in psychiatric disorders

Candidates of human lncRNAs associated with PDs were obtained from the lncRNA Disease database [116], a publicly available database of disease-associated lncRNAs. In addition, we performed a literature survey to identify further lncRNAs with PD association that are not listed in the lncRNA Disease database. In total, 26 human candidate lncRNAs were obtained (Additional file 1: Table S4 for IDs).

To annotate the orthologous lncRNAs in the other primate species, orthologous splice sites were first calculated in bonobo, chimpanzee, orangutan and rhesus macaque using the SpliceMap tool [22]. In addition to the splice sites, orthologous start and end sites were also calculated using both SpliceMap and BLASTN. A greedy approach was applied to retrieve the full set of orthologous, transcripts in BED12 format, based on the positions of starts, ends and splice sites (unpublished). To obtain the FASTA sequences from the BED12 coordinates, an in-house C program was used. Subsequently, local structure blocks were calculated, and the blocks with a *d* value below the threshold were submitted to the SSS-test. The same approach was used to identify the primate orthologs of Xist and HOTAIR. For SRA, we retrieved the orthologs from ENSEMBL’s BioMart.

## Additional file

Additional file 1 The Supplemental Material contains additional information on Methods and Data as well as additional Results. (PDF 1517 kb)

## Abbreviations

ENSG: Ensembl gene
FDR: False discovery rate
HAR1: Human accelerated region 1
HGNC: HUGO gene numenclature committee
HOTAIR: Hox antisense intergenic RNA
indel: Insertion or deletion
LINC: Long intergenic non-protein coding
lncRNA: Long non-coding RNA
MFE: Minimum free energy
MIAT: Myocardial infarction associated transcript
miRNA: microRNA
ncRNA: (small) non-coding RNA
ORF: Open reading frame
PD: Psychiatric disorder
SIX3-AS1: SIX3 antisense RNA 1
snoRNA: Small nucleolar RNA
SNP: Single nucleotide polymorphism
SRA: Steroid receptor RNA activator
SSS: Selection on the secondary structure
tRNA: Transfer RNA
TRPM2-AS: TRPM2 antisense RNA
Xist: X inactive-specific transcript
