# Supplementary material for: SSS-test: a novel test for detecting positive selection on RNA secondary structure
Source: BMC Bioinformatics. 2019 Mar 21;20:151. doi: 10.1186/s12859-019-2711-y (PMC6429701; doi:10.1186/s12859-019-2711-y)
Supplement: Supplementary file 1 — The Supplemental Material contains additional information on Methods and Data as well as additional Results. (PDF 1517 kb) [file 12859_2019_2711_MOESM1_ESM.pdf]

SSS-test: A Novel Test for Detecting Positive Selection on RNA Secondary Structure

Maria Beatriz Walter Costa, Christian Höner zu Siederdissen, Marko Dunjić, Peter F. Stadler and Katja Nowick

SUPPLEMENTAL MATERIAL

Structural divergence of ncRNA families

Looking for positively selected structures in a ncRNA family is only appropriate in a family with reasonable uniformity. A family that is too diverse could indicate different selective pressures, functionality through mechanisms other than structure or even incorrect orthology. Conversely, a uniform family points toward structural conservation and common selective pressures. In this regard the concept of structural divergence is important.

When applying the `SSS-test` on a few families, the scores along with a visual analysis of the secondary structures is ideal. The structures are very informative and their visual inspection yield valuable clues, such as forms and stability, which aid in choosing appropriate candidates. On the other hand screening entire databases is much more challenging, since visual inspection of all families is impossible. The `SSS-test` enables convenient filtering with a numeric score that indicates uniformity: the family divergence score d. The d score is the family's median species distance score d_s, which is calculated comparing the whole structural ensemble (dot plot files) of the species and its consensus, as explained in the main text.

As visual examples of a uniform and a non-uniform family, we have the snoRNA HACA76 and local block five of lncRNA blastnMacaque.Locus_222061

(Fig. 1). To best represent the probability ensemble visually, secondary structures can be used. The centroid structure is preferable rather than the most common used minimum free energy one, since the centroid represents the structure that is closest to all other possibilities.

snoRNA HACA76 (Fig. 1 left) is a uniform family with a possible candidate for positive selection. This family has a divergence score of $d = 0.1$ and selection scores indicating negative selection ($s = 0.0$) for all species, with the exception of Human, with a score that could indicate positive selection ($s = 15.8$). The visual profile of the structures of this family is clearly uniform, with only one structure (Human) standing out (Fig. 1 left). As a comparison, local block five of lncRNA blastnMacaque.Locus_222061 (Fig. 1 right) is a

non-uniform family, with a family divergence score of $d = 57.9$. Except for Orangutan, this block has intermediate and high selection scores for all species ($s > 5.0$). Importantly, in this particular case high selection scores unlikely point towards positive selection, rather than diverse functionality among the species, or as an alternative hypothesis, even incorrect orthology.

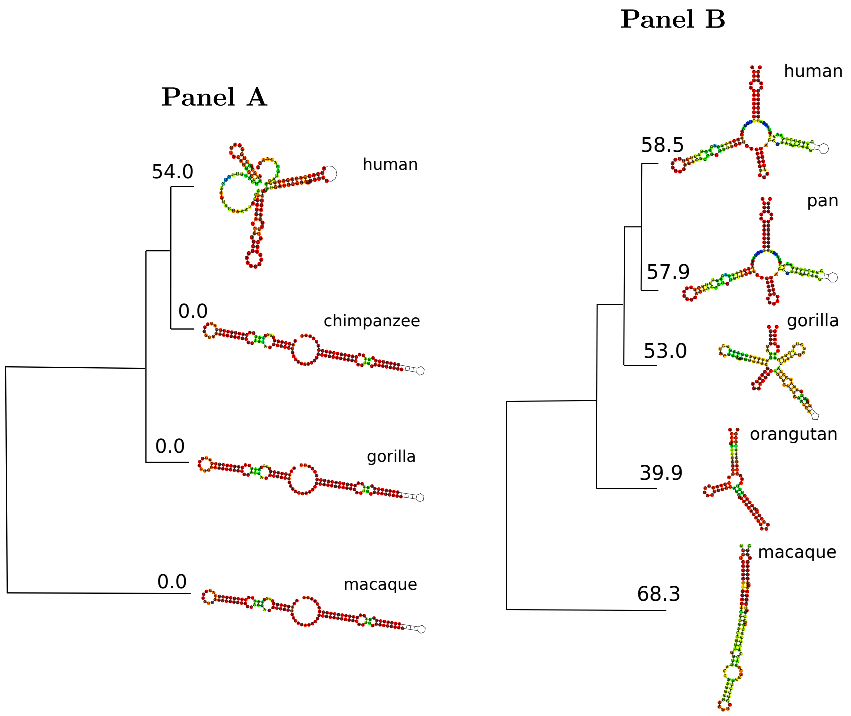

Figure 1: Centroid secondary structures of examples of uniform (Panel A) and a non-uniform (Panel B) ncRNA families. Panel A shows structures of snoRNA family HACA76, and Panel B shows structures of local block 5 of lncRNA blastnMacaque.Locus_2220619. Centroid structures are depicted here as visual indicators of the structural ensembles. snoRNA HACA76 (Panel A) is an example of a uniform family, with a clear trend of structure, except for one species (human). local block 5 of lncRNA blastnMacaque.Locus_2220619 (Panel B) is an example of a non-uniform family, with no clear trend of structure. The branching pattern was constructed according to the species phylogenetic tree, with the species structure distance score on the branches. Species distance score is defined by the structural distance between species and its consensus, normalized by the alignment length. Pan (Panel B) represents both chimpanzee and bonobo species, as described in (Necsulea et al., 2014).

Choice of an appropriate threshold for filtering non-uniform families in primate databases

Filtering out structurally non-uniform families is a reasonable first step for screening databases to look for positively selected structures. For that the family divergence score d of the `SSS-test` can be used, as discussed in the previous section. To choose an appropriate threshold for the primate databases used on this work, we visually inspected 12 families of ncRNAs (Table 1), with different divergence scores, ranging from $d = 0.0$ to $d = 65.0$.

Table 1: IDs of lncRNA local blocks used for visual analysis of family uniformity along with respective divergence scores (d) and outcome of visual analysis.

lncRNA family (*block ID*)	Divergence score (d)	Visual profile
blastnMouse.CUFF.110475 (*b-2*)	0.00	1 clear trend (no exception)
blastnMacaque.Locus_40302 (*b-4*)	5.00	1 clear trend (1 exception)
ENSG00000237166 (*b-4*)	7.50	1 clear trend (1 exception)
blastnPan.Locus_7625 (*b-1*)	10.00	1 clear trend (no exception)
ENSG00000243012 (*b-2*)	12.50	1 possible trend (1 exception)
blastnOpossum.Locus_375118 (*b-1*)	15.00	1 possible trend (1 exception)
ENSG00000256802 (*b-1*)	20.00	2 clear trends (no exception)
blastnMacaque.Locus_429229 (*b-1*)	25.00	1 possible trend (1 exception)
ENSG00000236466 (*b-1*)	35.00	2 possible trends
blastnPan.Locus_105878 (*b-1*)	45.05	no visuable trend
ENSG00000226526 (*b-3*)	55.05	no visuable trend
blastnPan.Locus_566 (*b-1*)	65.00	no visuable trend

For this analysis, the centroid structures of all species belonging to the family were taken into account along with the d scores (Fig. 2). The main criteria to classify a family as uniform was if one clear structural trend could be identified. All families with $d \leq 10.0$ have a clear trend, with no or only one exception of a different structure (Fig. 2 and Table 1). Families with $d > 10.0$ get increasingly more diverse, making it difficult to discern one clear structural trend (Fig. 2 and Table 1). A threshold of $d = 10.0$ was therefore chosen for this project.

According to this cutoff, families with divergence score $d \leq 10.0$ are considered uniform, while families with $d > 10.0$ are considered non-uniform and were filtered from further analysis.

Importantly, the choice of threshold may vary from project to project. The profiles observed in this work came from primates, which are phylogenetically very close. For different projects, with more distant or closer species, the threshold may be adapted to best fit the data. In addition, the candidates should be subjected to functional testing for confirmation of the predictions.

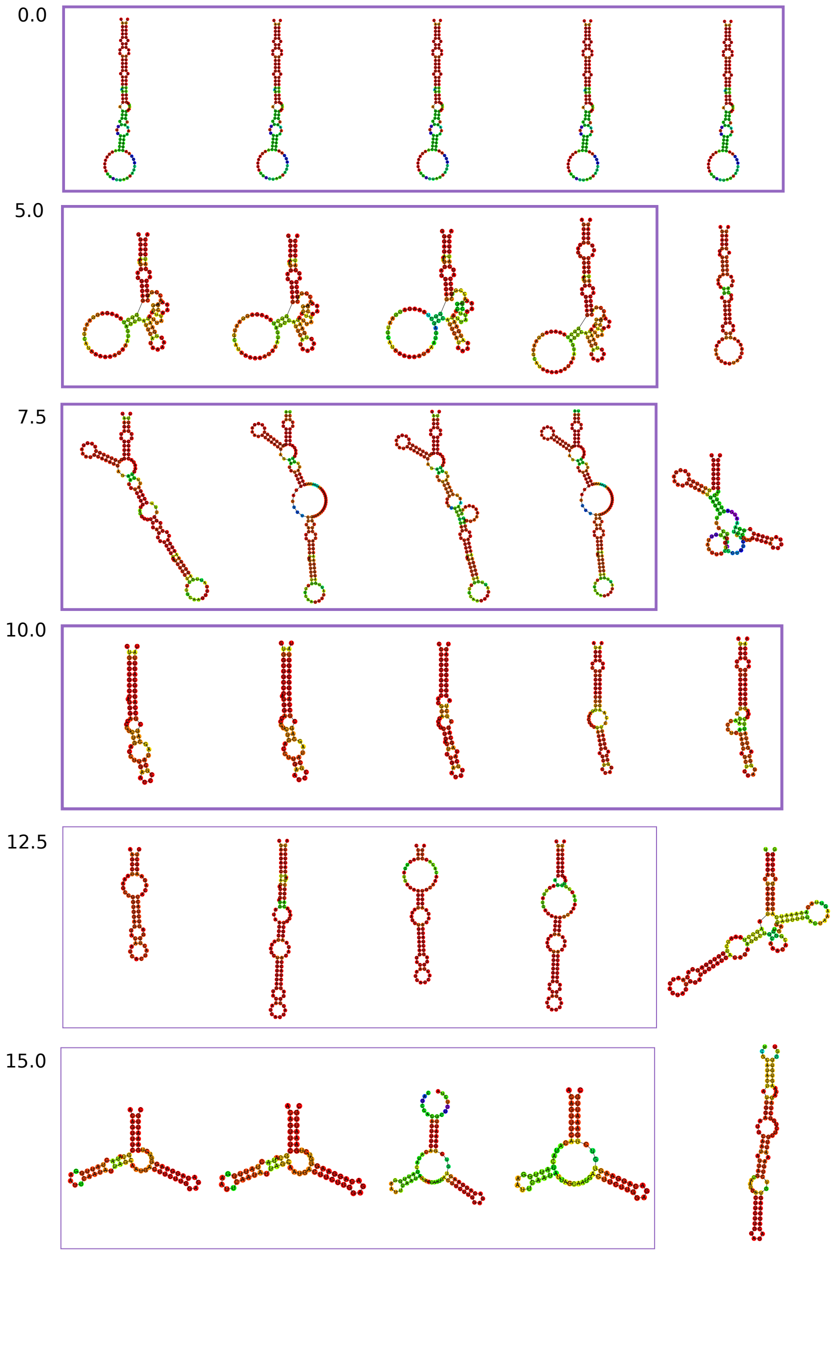
0.0
5.0
7.5
10.0
12.5
15.0

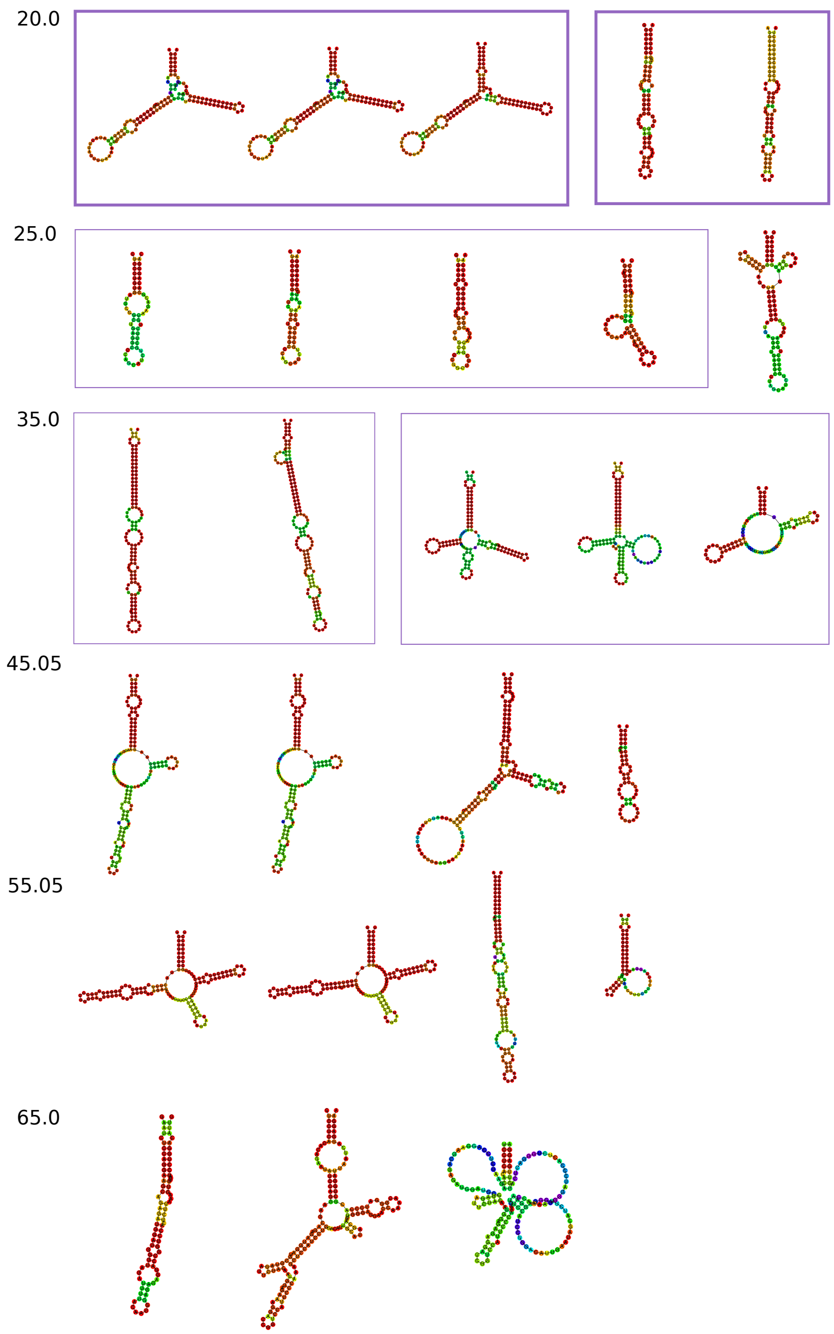

Figure 2: Centroid structures of lncRNA local block families with increasing divergence scores (d), $0.0 \leq d \leq 65.0$. The structural uniformity decreases with increasing d scores. Clear or possible structural trends were marked as thicker and thinner purple boxes respectively.

Choice of an appropriate threshold for positive and negative selection in primate databases

Analogously as for choosing a threshold for family divergence, human structures with different selection scores s were chosen for manual inspection. This led to the choice of an appropriate threshold for indicating positive and negative selection. For this analysis, 20 lncRNA families were chosen with different selection scores for the human structure, ranging from $s = 0.0$ to $s = 30.0$ (Table 2). The centroid structures of all species belonging to the family were taken into account along with the s scores of the human structure (Fig. 3). All considered families are low-divergent.

Table 2: IDs of lncRNA local sequences used for visual analysis of structures along with their respective selection scores (s) and outcome of visual analysis.

lncRNA (*block ID*)	Selection score (d)	Visual profile
blastnMacaque.Locus_61692 (*b-1*)	0.0	very similar form and stability
ENSG00000224711 (*b-5*)	1.0	very similar form and stability
blastnMacaque.Locus_62244 (*b-4*)	2.0	very similar form and stability
blastnMacaque.Locus_473621 (*b-6*)	3.0	similar form, higher stability
blastnPan.CUFF.296990 (*b-7*)	5.0	slight different form, lower stability
blastnMacaque.Locus_474656 (*b-2*)	9.0	clear different form, similar stability
Locus_193583 (*b-3*)	10.0	shorter form, higher stability
ENSG00000227509 (*b-7*)	13.3	clear different form, higher stability
blastnPan.Locus_17197 (*b-1*)	20.0	longer form, lower stability
blastnMacaque.Locus_210980 (*b-8*)	30.0	clear different form, higher stability

The two criteria for classifying the visual profile of the human structures were: (i) the similarity of their form in comparison to the other structures and (ii) their stability in comparison to the other structures. After careful analysis (Table 2 and Fig. 3), it could be observed that the human structures with $s \leq 3.0$ had very similar form and stability in relation to the other structures, which leads to the threshold of $s \leq 3.0$ to classify negative selection. Mixed profiles can be seen with scores $5.0 \leq s < 9.0$. With scores $s \geq 9.0$ there are

clear differences between the human structure and the others, in form and/or stability, which leads to the threshold of $s \geq 10.0$ to classify positive selection.

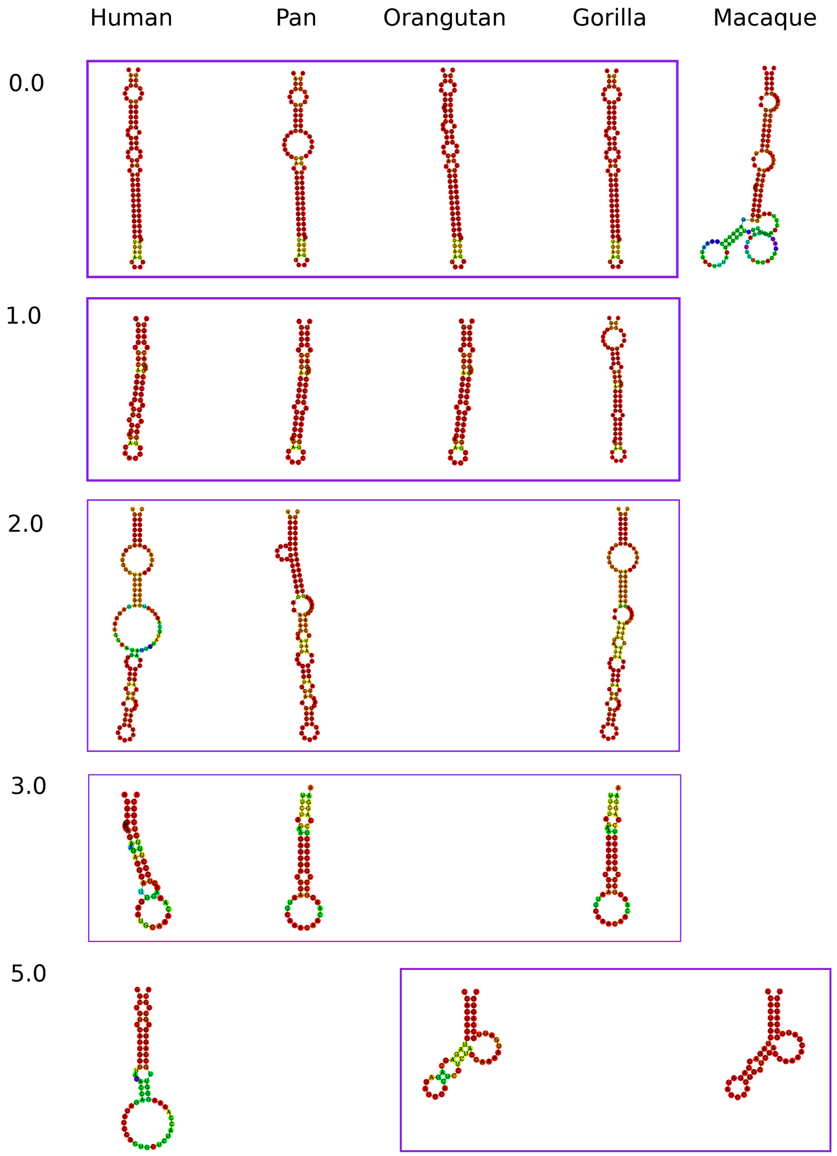
Human
Pan
Orangutan
Gorilla
Macaque
0.0
1.0
2.0
3.0
5.0

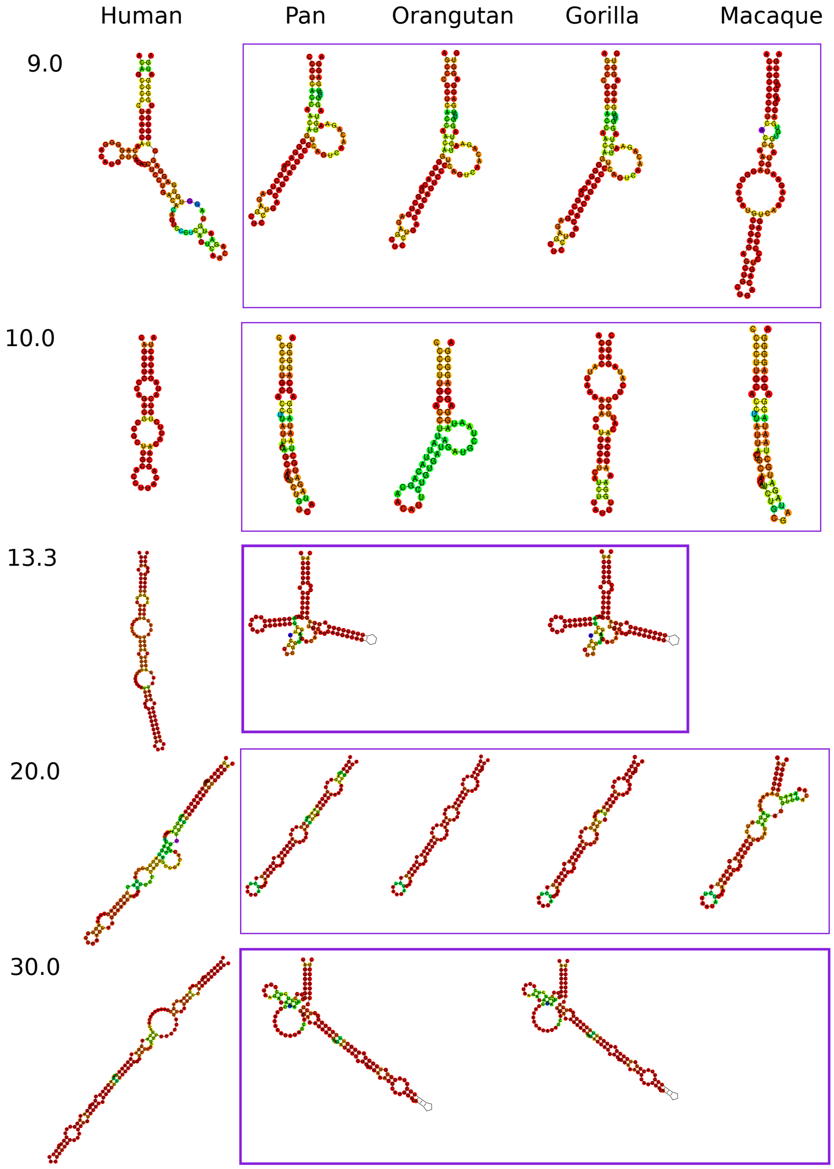

Figure 3: Local centroid structures of lncRNAs with increasing selection scores (s), $0.0 \leq s \leq 30.0$ for the human ones (left column).

Alternative Assessment of Positive Selection

Using the basic idea of the Ka/Ks-test, we classified single nucleotide changes into structurally conserved (sc) and structurally disruptive (sd). As in the SSS-test, we used `RNAsnp` p-values to discriminate between the types of sites. We then used the ratio of the fraction of observed structurally disruptive changes over all possible disruptive mutations (Ksd) and the fraction of observed structurally conservative changes over all possible conservative mutations (Ksc) as an indicator of selection pressures on the secondary structures (Ksc/Ksd approach). In the case of the positive selection control HAR, for example, we obtain for Ksd/Ksc: $(6/28)/(12/90) = 1.61$, and thus an indication of positive selection (if we consider the same thresholds as the Ka/Ks test, with scores $s > 1.0$ as indicative of positive selection).

The power of this approach however seems to be quite limited. First, it requires a reasonable number of changes for calculation purposes. This works well for HAR1, since it is the region in the entire human genome that accumulated the most human-specific changes ($c = 13$ according to our experiments, also accounting for compensatory bases). In contrast to that, the number of changes per lncRNA is usually rather small. In our analysed local blocks, the mean number of human-specific changes is $\bar{x} = 0.74$, with a variance of $\sigma^2 = 9$. In addition to that we found many false positive signals when analysing families by visual inspection.

We tested whether better estimates could be obtained by using a Poisson distribution for the expected number of substitutions parametrized by the expected change rate for a family, which is more appropriate when substitutions are sparse. This indeed improves the robustness, but still relies on the correctness of the classification of the sites, which in itself is not precise enough. This approach still led to many false positives. Hence, we abandoned this idea in

favor of using the evidence provided by `RNAsnp` as a quantitative rather than a categorical variable.

Using the Ksd/Ksc approach, we investigated a subset of the 15,443 families provided by Necsulea et al. (2014). This subset was composed of lncRNAs enriched in human-specific changes. 76 families showed in the primate alignment strong conservation among non-human primates and higher sequence divergence in humans (a similar situation to the HAR1). Applying the Ksd/Ksc approach to this collection resulted in 543 conserved local structures. Of these, 71 had sufficient orthologous substructures and 7 showed signs of positive selection in humans. For this analysis, no filter was applied in regards to family divergence.

From the positive selection candidates, two were also detected in the current approach of the test and five were not considered because of their family divergence, which is higher than the set threshold we applied in the new approach. Interestingly, the top scoring structure, which is part of the lncRNA H19X, received a score for the Ksd/Ksc approach of $s(\mathbf{H19Xsub2}) = 2.79$ and, like HAR1, has a significantly more stable secondary structure in human (Fig. 4).

Interestingly, this same structure received a positive selection score with the SSS-test ($s(\mathbf{H19Xsub2}) = 29.4$) and low score for the other species ($s \leq 1.2$). Although the family did not pass the divergence threshold ($d = 22.1$), we still suggest this structure as worthwhile to investigate, especially considering that the threshold serves for guiding purposes, and this family seems uniform enough.

Another one of the local structures found to be under positive selection in humans by the Ksd/Ksc approach was fundamentally different in humans, when compared to the other species. Visually, it did not seem to belong to the same group as the other structures. This brought the concern whether it was correctly annotated. To re-assess the orthology with a more robust approach, we used the Infernal suite (Nawrocki et al., 2009), by first building a covariance model for

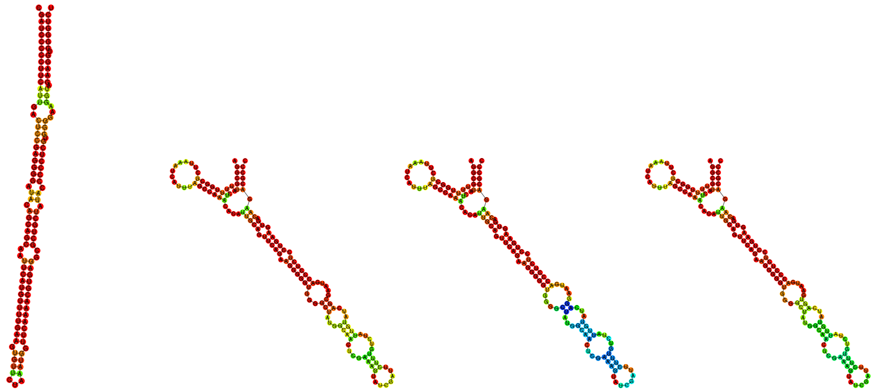

Figure 4: Local lncRNA structure predictions for **H19Xsub2**: (left to right): human, gorilla, orangutan and pan. The human structure received a positive selection signal with the Ksd/Ksc approach, as well as the approach of the SSS-test. The colours of the bases are assigned according to their pairing frequency in the structure's ensemble. Shades of red occur in $\geq 90\%$ of the ensemble, shades of green/yellow denote increasing probabilities from $\geq 50\%$. For unpaired bases, shades of red denote increasing unpairedness.

the non-human primate structures, then calibrating it and finally searching it in the entire human genome. Interestingly, the single hit maps to an intronic part of the orthologous transcripts, suggesting that the human lncRNA may have lost one exon (Fig. 5). The annotated gene structure is drastically different in the different primates. While human and macaque have only two exons, chimpanzee/bonobo and gorilla have five, and orangutan features six introns, suggesting a rapid turnover of gene structure in this lncRNA gene.

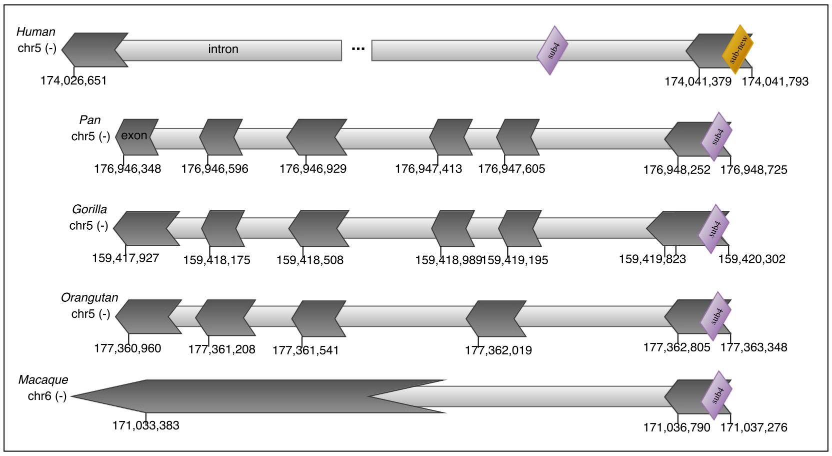

Figure 5: Revised orthology for local structure 4 of lncRNA **CUFF.464429**. The initially classified human **CUFF.464429sub4**, depicted in yellow, initially received a positive selection signal with the Ksd/Ksc approach. This structure was re-classified to a new orthologous group and the revised human orhologous of **CUFF.464429sub4**, depicted in purple, was found by using the non-human primate structures as a search model. More details in the supplemental text. Coordinates refer to assemblies of ENSEMBL v62 as described in (Necsulea et al., 2014).

lncRNAs involved in psychiatric disorders

Coordinates of the analysed transcripts (Table 4) were obtained from the UCSC table browser `https://genome.ucsc.edu/cgi-bin/hgTables` in BED12 format for the *Homo sapiens* genome assembly version hg38. The ortholog lncRNAs were calculated for the following primates (and assembly versions): *Pan paniscus* (panPan1), *Pan troglodytes* (panTro4), *Pongo abelii* (ponAbe2), and *Macaca mulatta* (rheMac3).

Indel impact on structure

One of the challenges of detecting positive selection on ncRNA structures is to account for the structural impact of indels. To assess indel impact on ncRNA structures, we build a framework to simulate gap evolution (Fig. 6). Given an input ncRNA (the ancestral structure), it evolves the sequence inputing gaps of size n (defined by the user) in a window-based manner, from the first base to the last. It assesses and outputs the structural impact of the gap in all positions using the RNAforester tool (Höchsmann, 2005).

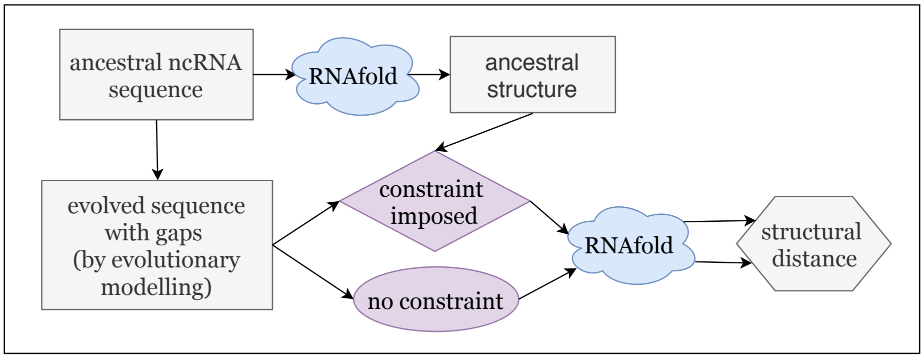

Figure 6: Gap analysis framework to asssess the structural impact of gaps in ncRNA structures.

Importantly, RNA structure comparison cannot be treated like the problem of sequence comparison, for instance. In sequence comparison, there is only one layer of complexity, the order of the elements, or bases, while in structure comparison, there is another layer that must be accounted for, which is the secondary dimension of the structure. RNAforester implements structural tree alignments, pointing out their dissimilarities, which is convenient for calculating indel impact on a structure. In addition, structures of different lengths can also be compared with this tool.

Using this idea and applying rank statistics, we accounted for the structural impact of observed indels in the SSS-test (more information on the main text).

We also applied the gap analysis framework (Fig. 6) on biological RNAs (one example in Fig. 7), to check if the length of the gap was important for the structural impact. The sequence of the tRNA used in the example is:

```
>tRNA
UUUGGGUGUAUAGCUCAGUUGGUAGAGCAUUGGGCUUUUAACCUAAUGGUCGCAGGUUCA
AGUCCUGCUAUACCCACCA
```

We performed the same experiment in 12 other biological RNAs and noticed that the length of the gap did not matter for the impact, but rather its location. If the gap overlaps a paired region, its impact on structure is usually high and comparable with different gap lengths (Fig. 7). Conversely, if the gap does not overlap a paired region, its impact is usually zero (Fig. 7).

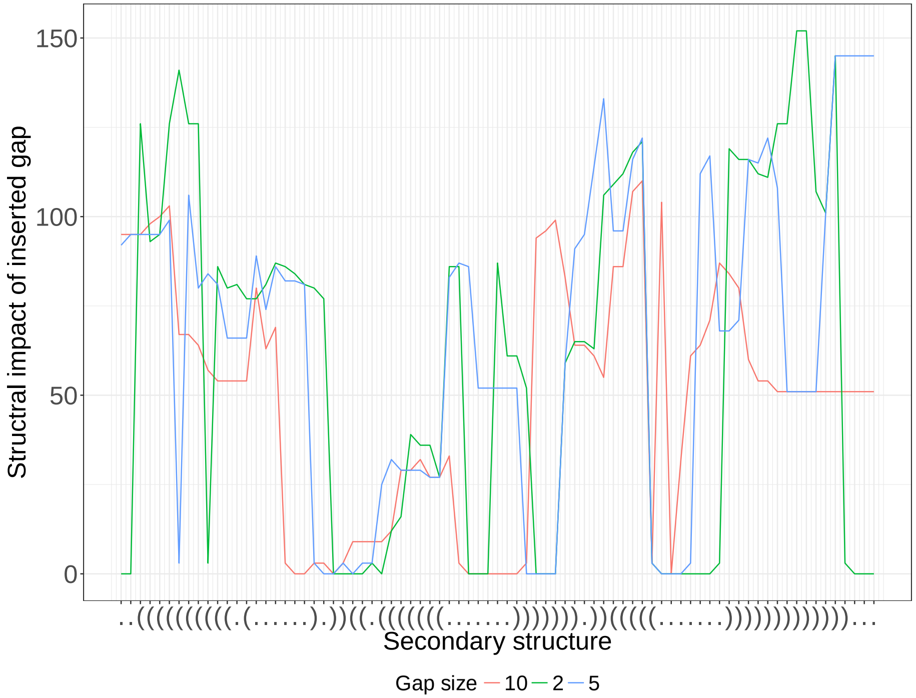

Figure 7: Structural impact of inserted gaps in a tRNA structure. x axis: tRNA base positions from 1 to 78, y axis: structural impact of the inserted gap at position x. Blue line: gap of size 2, orange line: gap of size 5, red line: gap of size 10. As a reference, the dot-bracket structure of the tRNA MFE structure is below the sequence. Note that the impact is zero when the gap is located in an unpaired region (denoted by dots), and it is higher whenever the gap overlaps a paired region. This behaviour is independent of the gap size.

SSS-test implementation and usage

The SSS-test was implemented as a bash script `SSS.sh` that calls separate perl scripts executing specific modules. These were used for all experiments cited in the main paper and can be downloaded along with a README tutorial file at: `http://www.bioinf.uni-leipzig.de/Software/SSS-test/`.

The `SSS.sh` can be run in the command line as:

```
SSS.sh -i <FASTA_FILE> -f <FILE_FORMAT> -s <Yes/No>
```

with -i indicating input file, -f format (fasta or aligned) and -s if the user wants the secondary structures to be saved or not in a subfolder.

The following tools are used internally, and must be installed beforehand:

- RNAsnp (Sabarinathan et al., 2013)
- muscle (Edgar, 2004)
- Vienna RNA package (Lorenz et al., 2011)
- Bio::AlignIO `http://search.cpan.org/dist/BioPerl/Bio/AlignIO.pm`
- Statistics::R `http://search.cpan.org/~gmpassos/Statistics-R-0.02/lib/Statistics/R.pm`

Supplemental Tables and Figures

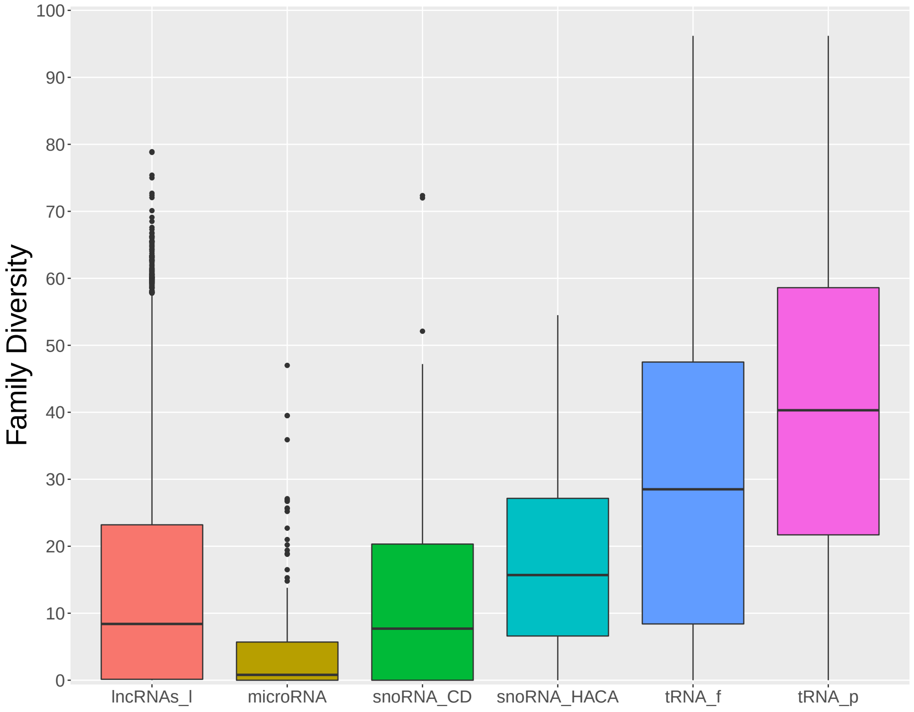

Figure 8: Family structural divergence of different classes of ncRNA, x axis: ncRNA class, y axis: family divergence. From left to right: lncRNA local blocks, microRNAs, CD box snoRNAs, HACA box snoRNAs, functional tRNAs and pseudo tRNAs. Family divergence is defined as the median divergence score of the family (more details in the supplemental text).

Table 3: Summary of the selection analysis of the lncRNA families provided by (Necsulea et al., 2014).

Species	Initial set	orthologous lncRNA families	local blocks (total)	local blocks ($species \geq 3$)	Family Divergence	
					low ($d \leq 10$)	high ($d > 10$)
Human	14,682					
Pan	14,654					
Orangutan	13,756	15,443	87,613	19,408	10,396	9,012
Gorilla	14,258				53,57%	46,43%
Macaque	15,280					

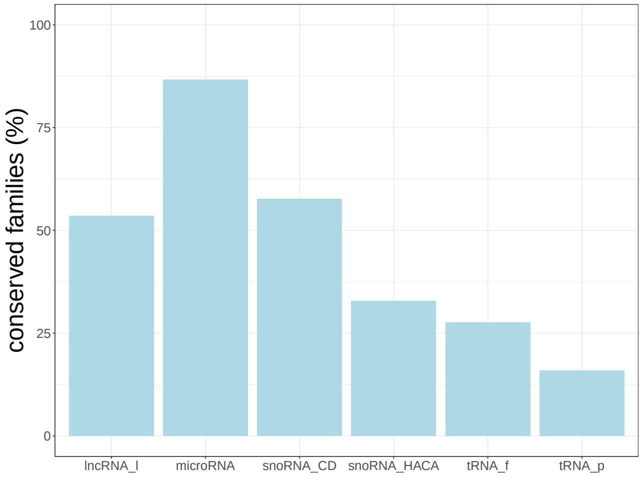

Figure 9: Conservation overview of ncRNA classes: family conservation. x axis: classes; y axis: percentage of conserved families ($d \leq 10.0$). From left to right, the classes correspond to: (i) lncRNA local structures, (ii) microRNAs, (iii) CD box snoRNAs, (iv) HACA box snoRNAs, (v) functional tRNAs and (vi) pseudo tRNAs.

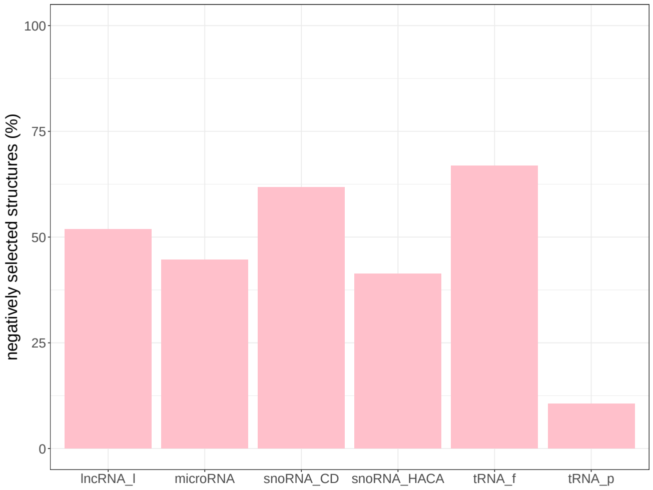

Figure 10: Conservation overview of ncRNA classes: negative selection. x axis: classes; y axis: percentage of structures in the most conserved bin ($s = 0.0$), indicating strong negative selection. Assessment of negative selection was made only within conserved families ($d \leq 10.0$). From left to right, the classes correspond to: (i) lncRNA local structures, (ii) microRNAs, (iii) CD box snoRNAs, (iv) HACA box snoRNAs, (v) functional tRNAs and (vi) pseudo tRNAs.

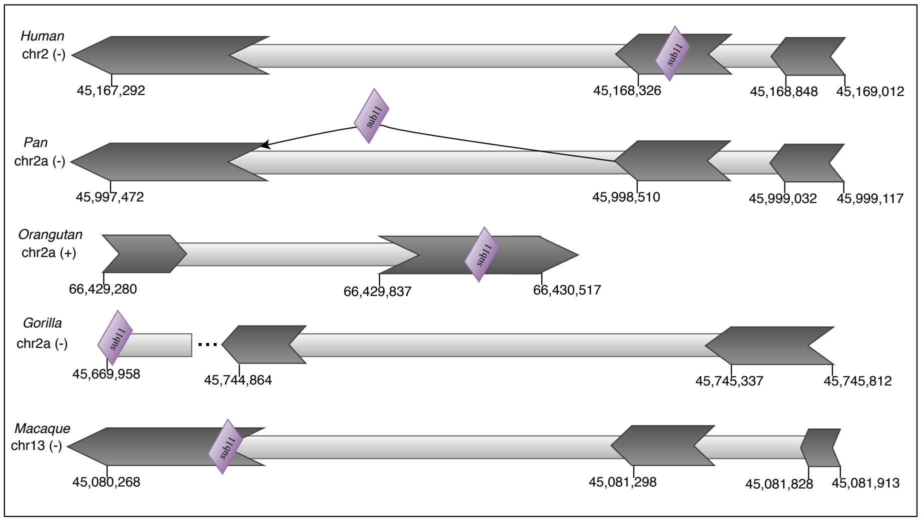

Figure 11: Orthologs of the lncRNA SIX3-AS1 in five primates with a conserved local structure. Local structure *sub11*, denoted in purple, show signs of positive selection in humans and negative selection in the other species. This local structure is present in all five primates, being located in the exons of human, orangutan and macaque, in the spliced transcript of pan, between two exons and outside of the reported locus in gorilla, in a region with no other annotated elements. Introns are depicted as light grey rectangles and exons as dark grey arrows. Coordinates are given in accordance to (Necsulea et al., 2014) for the assemblies of ENSEMBL v62.

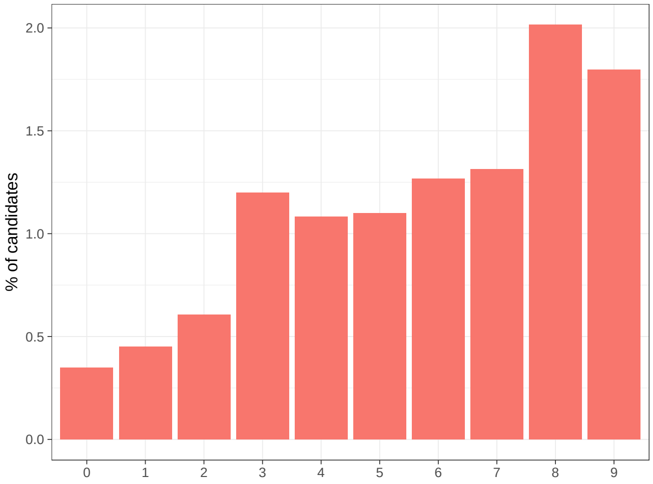

Figure 12: Tissue specificity of the human lncRNAs that are candidates for having structures under positive selection. x axis: number of tissues of expression (with zero refering to no detectable expression), y axis, percentage of candidates in each x group.

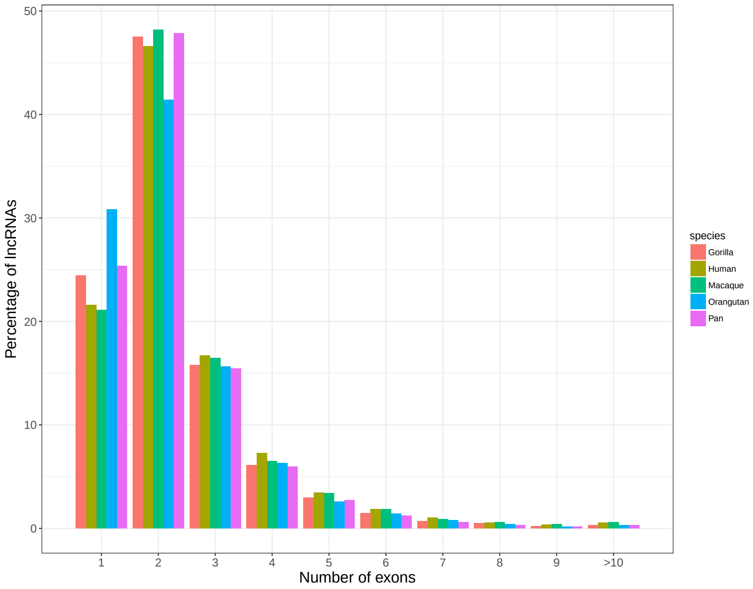

Figure 13: Exon size distribution of the analysed lncRNA dataset provided by (Necsulea et al., 2014). The five primates show similar distribution, as expected. Most lncRNAs have two exons and few have more than five exons. A considerable percentage of lncRNAs have only one exon.

Table 4: List of lncRNAs implicated in psychiatric disorders (PD).

ENSEMBL Transcript ID	Gene Name	PDs	References
ENST00000637926.1	GDNF-AS1	Alzheimer's disease	(Airavaara et al., 2011)
ENST00000634594.1	MIR137HG	Schizophrenia	(Ripke et al., 2011) (Wright et al., 2017)
ENST00000618857.1	BACE1-AS	Alzheimer's disease	(Modarresi et al., 2011)
ENST00000613780.4	MIAT (Gomafu)	Schizophrenia; Alzheimer's disease; Substance dependence	(Barry et al., 2014)(Rao et al., 2015) (Michelhaugh et al., 2011) (Jiang et al., 2016)
ENST00000594922.5	FMR1-AS1	Fragile X syndrome	(Ladd et al., 2007)
ENST00000551288.5	EMX2OS	Substance dependence	(Michelhaugh et al., 2011)
ENST00000534336.1	MALAT1 (NEAT2)	Substance dependence	(Michelhaugh et al., 2011) (Kryger et al., 2012)
ENST00000532226.1	TRAF3IP2-AS1 (C6UAS)	Schizophrenia	(Morelli et al., 2000)
ENST00000522771.7	MEG3	Substance dependence	(Michelhaugh et al., 2011)
ENST00000501122.2	NEAT1	Substance dependence	(Michelhaugh et al., 2011)
ENST00000499008.7	BDNF-AS	Autism spectrum disorders	(Modarresi et al., 2012)
ENST00000498731.5	SOX2-OT	Alzheimer's disease	(Arisi et al., 2011)
ENST00000456775.1	ST7-OT1	Autism spectrum disorders	(Vincent et al., 2002)
ENST00000456577.5	ST7-OT2	Autism spectrum disorders	(Vincent et al., 2002)
ENST00000455399.1	PTCHD1-AS	Autism spectrum disorders	(Noor et al., 2010)
ENST00000452629.1	LINC02151	Major depressive disorder	(Cui et al., 2016)
ENST00000566208.1	LINC02152	Major depressive disorder	(Cui et al., 2016)
ENST00000522604.1	LINC02153	Major depressive disorder	(Cui et al., 2016)
ENST00000448407.1	DAOA-AS1	Schizophrenia; Bipolar disorder	(Hattori et al., 2003) (Chumakov et al., 2002)
ENST00000439725.5	H19	Substance dependence	(Ouko et al., 2009)
ENST00000437681.1	SNHG3	Alzheimer's disease	(Arisi et al., 2011)
ENST00000428597.5	CDKN2B-AS1	Alzheimer's disease	(Züchner et al., 2008)
ENST00000421378.2	LINC00271	Schizophrenia	(Amann-Zalcenstein et al., 2006)
ENST00000429268.1	SHANK2-AS2	Autism spectrum disorders	(Wang et al., 2015)
ENST00000413238.1	LINC00689	Autism spectrum disorders	(Parikshak et al., 2016)
ENST00000397750.7	ST7-OT4	Autism spectrum disorders	(Vincent et al., 2002)

(a) Human

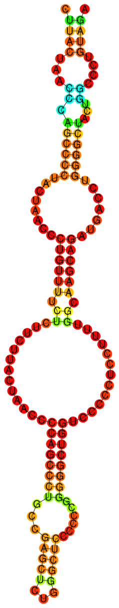

(b) Bonobo

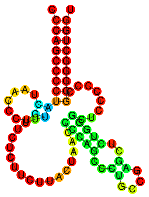

(c) Chimpanzeee

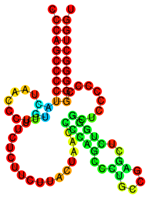

(d) Orangutan

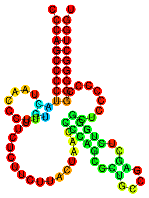

(e) Rhesus macaque

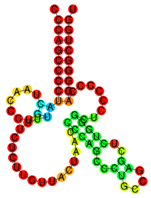

Figure 14: Comparison of **MIATsub31** MFE structures. **MIATsub31** local structure is highly conserved among non-human primates, and different in humans, with a longer more stable structure, and a signal for positive selection.

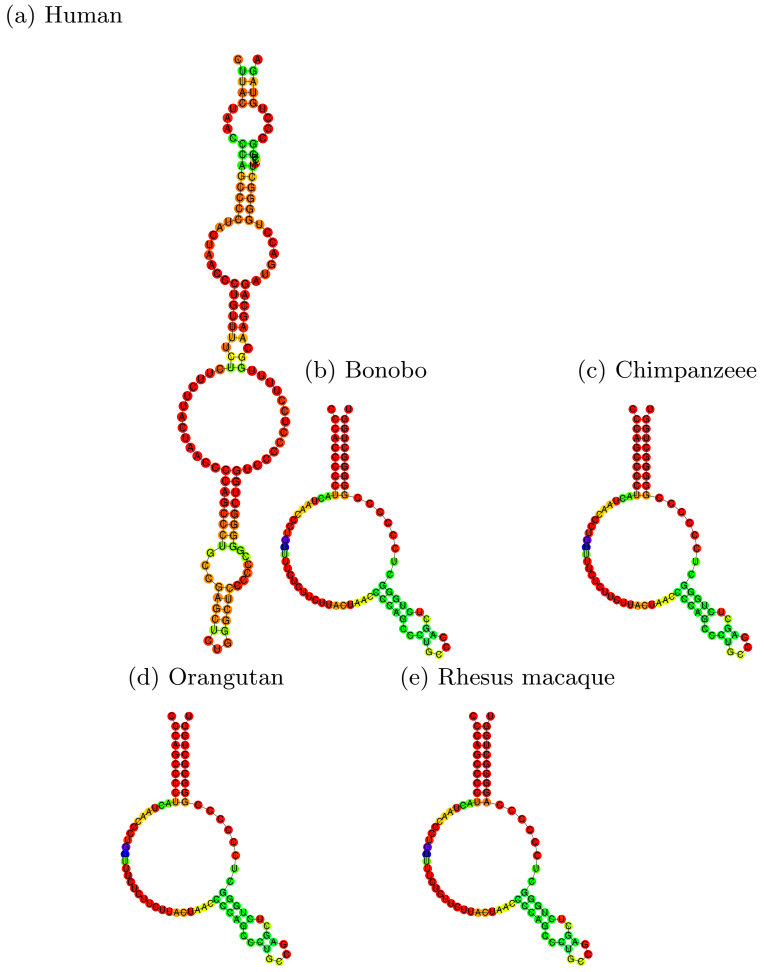

Figure 15: Comparison of **MIATsub31** centroid structures. **MIATsub31** local structure is highly conserved among non-human primates, and different in humans, with a longer more stable structure, and a signal for positive selection.

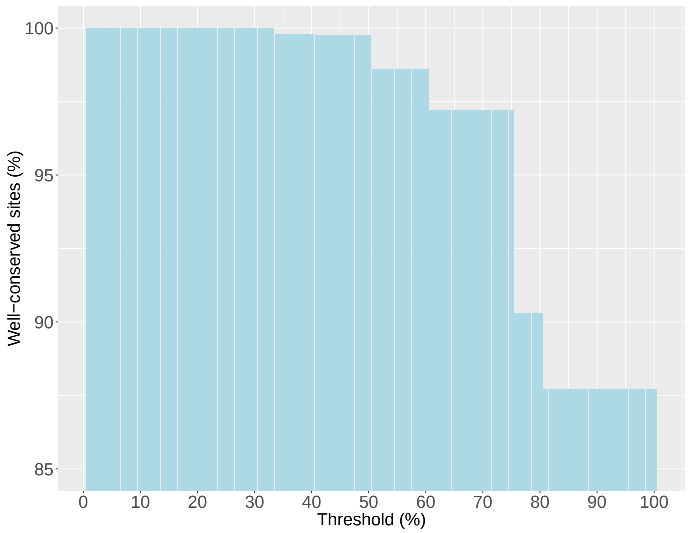

Figure 16: Percentage of sites that reach the threshold to be considered well-conserved, per different thresholds of what consititutes a well-conserved site from 1 to 100%. The default of the `SSS-test` is a 60% threshold, which leads to 98.6% of the sites being well-conserved.

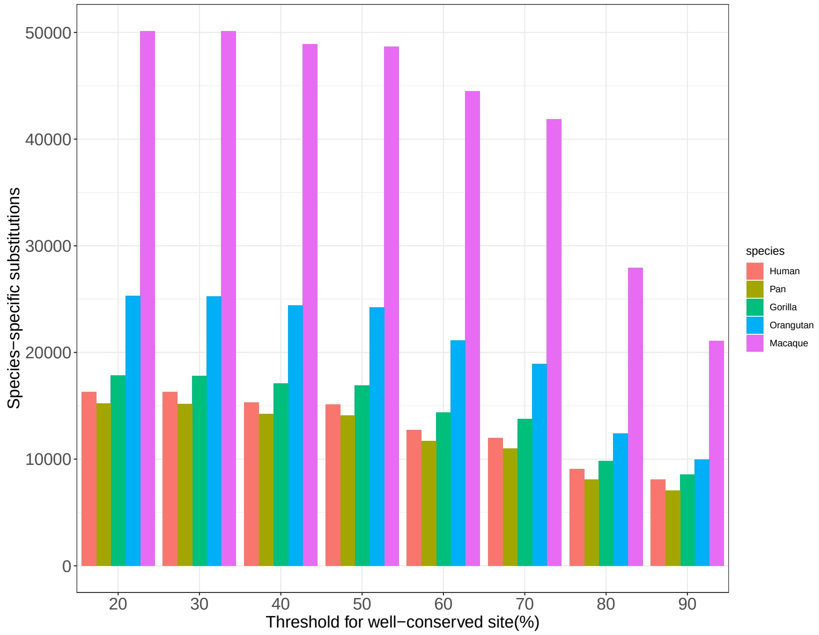

Figure 17: Number of species specific substitutions per different thresholds of well-conserved sites. The default of the `SSS-test` is a 60% threshold.

Table 5: Number of species-specific substitutions (compensatory sites excluded).

Species	Species specific substitutions	Total number of sites	Average species specific substitutions (%)
Human	11,741	1,517,490	0.77
Pan	10,816	1,460,758	0.74
Gorilla	13,309	1,355,782	0.98
Orangutan	19,027	1,199,603	1.57
Macaque	41,280	1,086,974	3.80

Table 6: Compensatory sites (substitutions) per species calculated with the `SSS-test` with the default threshold of 60% for well-conserved sites.

Species	Compensatory substitutions	Total substitutions (compensatory excluded)	Sum	Compensatory substitutions (%)
Human	980	11,741	12,721	7.7
Pan	890	10,816	11,707	7.6
Gorilla	1,090	13,309	14,399	7.6
Orangutan	2,091	19,027	21,118	9.9
Macaque	3,202	41,280	44,482	7.2

References

Airavaara, M., Pletnikova, O., Doyle, M. E., Zhang, Y. E., Troncoso, J. C., and Liu, Q.-R. (2011). Identification of novel GDNF isoforms and cis-antisense GDNFOS gene and their regulation in human middle temporal gyrus of Alzheimer disease. *Journal of Biological Chemistry*, 286(52):45093–45102.

Amann-Zalcenstein, D., Avidan, N., Kanyas, K., Ebstein, R. P., Kohn, Y., Hamdan, A., Ben-Asher, E., Karni, O., Mujaheed, M., Segman, R. H., et al. (2006). AHI1, a pivotal neurodevelopmental gene, and C6orf217 are associated with susceptibility to schizophrenia. *European Journal of Human Genetics*, 14(10):1111.

Arisi, I., D'Onofrio, M., Brandi, R., Felsani, A., Capsoni, S., Drovandi, G., Felici, G., Weitschek, E., Bertolazzi, P., and Cattaneo, A. (2011). Gene

expression biomarkers in the brain of a mouse model for alzheimer's disease: mining of microarray data by logic classification and feature selection. *Journal of Alzheimer's Disease*, 24(4):721–738.

Barry, G., Briggs, J., Vanichkina, D., Poth, E., Beveridge, N., Ratnu, V., Nayler, S., Nones, K., Hu, J., Bredy, T., et al. (2014). The long non-coding RNA Gomafu is acutely regulated in response to neuronal activation and involved in schizophrenia-associated alternative splicing. *Molecular psychiatry*, 19(4):486.

Chumakov, I., Blumenfeld, M., Guerassimenko, O., Cavarec, L., Palicio, M., Abderrahim, H., Bougueleret, L., Barry, C., Tanaka, H., La Rosa, P., et al. (2002). Genetic and physiological data implicating the new human gene G72 and the gene for D-amino acid oxidase in schizophrenia. *Proceedings of the National Academy of Sciences*, 99(21):13675–13680.

Cui, X., Sun, X., Niu, W., Kong, L., He, M., Zhong, A., Chen, S., Jiang, K., Zhang, L., and Cheng, Z. (2016). Long non-coding RNA: Potential diagnostic and therapeutic biomarker for major depressive disorder. *Medical science monitor: international medical journal of experimental and clinical research*, 22:5240.

Edgar, R. C. (2004). MUSCLE: multiple sequence alignment with high accuracy and high throughput. *Nucleic acids research*, 32(5):1792–1797.

Hattori, E., Liu, C., Badner, J. A., Bonner, T. I., Christian, S. L., Maheshwari, M., Detera-Wadleigh, S. D., Gibbs, R. A., and Gershon, E. S. (2003). Polymorphisms at the G72/G30 gene locus, on 13q33, are associated with bipolar disorder in two independent pedigree series. *The American Journal of Human Genetics*, 72(5):1131–1140.

Höchsmann, M. (2005). The tree alignment model: algorithms, implementations

and applications for the analysis of RNA secondary structures. *Bielefeld University.*

Jiang, Q., Shan, K., Qun-Wang, X., Zhou, R.-M., Yang, H., Liu, C., Li, Y.-J., Yao, J., Li, X.-M., Shen, Y., et al. (2016). Long non-coding RNA-MIAT promotes neurovascular remodeling in the eye and brain. *Oncotarget*, 7(31):49688.

Kryger, R., Fan, L., Wilce, P. A., and Jaquet, V. (2012). MALAT-1, a non protein-coding RNA is upregulated in the cerebellum, hippocampus and brain stem of human alcoholics. *Alcohol*, 46(7):629–634.

Ladd, P. D., Smith, L. E., Rabaia, N. A., Moore, J. M., Georges, S. A., Hansen, R. S., Hagerman, R. J., Tassone, F., Tapscott, S. J., and Filippova, G. N. (2007). An antisense transcript spanning the CGG repeat region of FMR1 is upregulated in premutation carriers but silenced in full mutation individuals. *Human molecular genetics*, 16(24):3174–3187.

Lorenz, R., Bernhart, S. H., Zu Siederdissen, C. H., Tafer, H., Flamm, C., Stadler, P. F., and Hofacker, I. L. (2011). ViennaRNA package 2.0. *Algorithms for Molecular Biology*, 6(1):1.

Michelhaugh, S. K., Lipovich, L., Blythe, J., Jia, H., Kapatos, G., and Bannon, M. J. (2011). Mining affymetrix microarray data for long non-coding RNAs: altered expression in the nucleus accumbens of heroin abusers. *Journal of neurochemistry*, 116(3):459–466.

Modarresi, F., Faghihi, M. A., Lopez-Toledano, M. A., Fatemi, R. P., Magistri, M., Brothers, S. P., Van Der Brug, M. P., and Wahlestedt, C. (2012). Inhibition of natural antisense transcripts in vivo results in gene-specific transcriptional upregulation. *Nature biotechnology*, 30(5):453.

Modarresi, F., Faghihi, M. A., Patel, N. S., Sahagan, B. G., Wahlestedt, C.,

and Lopez-Toledano, M. A. (2011). Knockdown of BACE1-AS nonprotein-coding transcript modulates beta-amyloid-related hippocampal neurogenesis. *International Journal of Alzheimer's Disease*, 2011.

Morelli, C., Magnanini, C., Mungall, A. J., Negrini, M., and Barbanti-Brodano, G. (2000). Cloning and characterization of two overlapping genes in a subregion at 6q21 involved in replicative senescence and schizophrenia. *Gene*, 252(1):217–225.

Nawrocki, E. P., Kolbe, D. L., and Eddy, S. R. (2009). Infernal 1.0: inference of RNA alignments. *Bioinformatics*, 25(10):1335–1337.

Necsulea, A., Soumillon, M., Warnefors, M., Liechti, A., Daish, T., Zeller, U., Baker, J. C., Grützner, F., and Kaessmann, H. (2014). The evolution of lncRNA repertoires and expression patterns in tetrapods. *Nature*, 505:635–640.

Noor, A., Whibley, A., Marshall, C. R., Gianakopoulos, P. J., Piton, A., Carson, A. R., Orlic-Milacic, M., Lionel, A. C., Sato, D., Pinto, D., et al. (2010). Disruption at the PTCHD1 locus on Xp22. 11 in autism spectrum disorder and intellectual disability. *Science translational medicine*, 2(49):49ra68–49ra68.

Ouko, L. A., Shantikumar, K., Knezovich, J., Haycock, P., Schnugh, D. J., and Ramsay, M. (2009). Effect of alcohol consumption on CpG methylation in the differentially methylated regions of H19 and IG-DMR in male gametes—implications for fetal alcohol spectrum disorders. *Alcoholism: Clinical and Experimental Research*, 33(9):1615–1627.

Parikshak, N. N., Swarup, V., Belgard, T. G., Irimia, M., Ramaswami, G., Gandal, M. J., Hartl, C., Leppa, V., de la Torre Ubieta, L., Huang, J., et al. (2016). Genome-wide changes in lncRNA, splicing, and regional gene expression patterns in autism. *Nature*, 540(7633):423.

Rao, S.-Q., Hu, H.-L., Ye, N., Shen, Y., and Xu, Q. (2015). Genetic variants in long non-coding RNA MIAT contribute to risk of paranoid schizophrenia in a chinese Han population. *Schizophrenia research*, 166(1):125–130.

Ripke, S., Sanders, A. R., Kendler, K. S., Levinson, D. F., Sklar, P., Holmans, P. A., Lin, D.-Y., Duan, J., Ophoff, R. A., Andreassen, O. A., et al. (2011). Genome-wide association study identifies five new schizophrenia loci. *Nature genetics*, 43(10):969.

Sabarinathan, R., Tafer, H., Seemann, S. E., Hofacker, I. L., Stadler, P. F., and Gorodkin, J. (2013). `RNAsnp`: Efficient detection of local RNA secondary structure changes induced by SNPs. *Hum. Mut.*, 34:546–556.

Vincent, J. B., Petek, E., Thevarkunnel, S., Kolozsvari, D., Cheung, J., Patel, M., and Scherer, S. W. (2002). The RAY1/ST7 tumor-suppressor locus on chromosome 7q31 represents a complex multi-transcript system. *Genomics*, 80(3):283–294.

Wang, Y., Zhao, X., Ju, W., Flory, M., Zhong, J., Jiang, S., Wang, P., Dong, X., Tao, X., Chen, Q., et al. (2015). Genome-wide differential expression of synaptic long noncoding RNAs in autism spectrum disorder. *Translational psychiatry*, 5(10):e660.

Wright, C., Gupta, C., Chen, J., Patel, V., Calhoun, V. D., Ehrlich, S., Wang, L., Bustillo, J., Perrone-Bizzozero, N., and Turner, J. (2017). Polymorphisms in MIR137HG and microRNA-137-regulated genes influence gray matter structure in schizophrenia. *Translational psychiatry*, 6(2):e724.

Züchner, S., Gilbert, J., Martin, E., Leon-Guerrero, C., Xu, P.-T., Browning, C., Bronson, P., Whitehead, P., Schmechel, D., Haines, J., et al. (2008). Linkage and association study of late-onset alzheimer disease families linked to 9p21. 3. *Annals of human genetics*, 72(6):725–731.
